# Biofilm-Forming Capacity of ESBL *Escherichia coli* and Influential Factors and Efficacy of Essential Oils in Inhibition and Disruption

**DOI:** 10.3390/antibiotics15101022

**Published:** 2026-10-15

**Authors:** Anita Seres-Steinbach, Péter Szabó, Ágnes Solti-Hodován, Viktória Lilla Balázs, Krisztián Bányai, György Schneider

**Affiliations:** 1 Department of Medical Microbiology and Immunology, Medical School, University of Pécs, H-7624 Pécs, Hungary; seres-steinbach.anita@edu.pte.hu (A.S.-S.); hodovan.agnes2@pte.hu (Á.S.-H.); 2 Environmental Analytical and Geoanalytical Research Group, Szentágothai Research Center, Ifjúság Str. 20., H-7624 Pécs, Hungary; sz.piiit01@gmail.com; 3 Department of Biophysics, Medical School, University of Pécs, Szigeti Str. 12., H-7624 Pécs, Hungary; 4 Department of Pharmacognosy, Faculty of Pharmacy, University of Pécs, H-7624 Pécs, Hungary; balazs.viktoria@gytk.pte.hu; 5 Department of Medical Biology, Medical School, University of Pécs, H-7624 Pécs, Hungary; banyai.krisztian@aok.pte.hu or banyai.krisztian@univet.hu; 6 Department of Pharmacology and Toxicology, University of Veterinary Medicine, H-1078 Budapest, Hungary

**Keywords:** essential oils, ESBL-producing *E. coli*, biofilm, bioautography

## Abstract

Background/Objectives: The presence of extended-spectrum β-lactamase-producing *Escherichia coli* (ESBL-producing *E. coli*) is a major global concern as it causes hard-to-treat infections that can sometimes be life-threatening. Methods: Using the drop plate method, we examined which essential oils (EOs) were effective against the tested strains. After mapping the general biofilm-forming affinity of the isolates, we attempted to degrade the biofilm using the EOs. Results: Certain EOs can kill ESBL-producing *E. coli*, inhibit biofilm formation, and degrade mature biofilms. The drop plate method showed that 50 (83%) and 54 (90%) of the 60 tested EOs were effective against 20 different human and porcine ESBL-producing *E. coli* isolates when applied at 1% and 5%, respectively. The most effective ESBL-producing *E. coli* isolate (23R20) was used for anti-biofilm testing. The temperature of 37 °C was most effective in inhibiting and degrading biofilms, although this was markedly influenced by the carrier medium. Ajowan, cinnamon, oregano, and thyme had outstanding effects in both tests, inhibiting and degrading biofilms by 99%, respectively. Morphological differences (e.g., deformation, elongation or shortening of cells, amorphous structures, and ‘ghost’/perforated cells) were noted in the 23R20 *E. coli* cells exposed to ajowan, cinnamon, oregano, and thyme. Conclusions: Ajowan, cinnamon, oregano, and thyme were the most effective biofilm-degrading and -inhibitory EOs.

## 1. Introduction

Extended-spectrum β-lactamases (ESBLs) are defined as enzymes produced by certain bacteria that are able to hydrolyse several new-generation β-lactam antibiotics, such as ceftazidime, ceftriaxone, cefotaxime, oxyimino-monobactam, and other β-lactam antibiotics, and thus make antibacterial therapy a real challenge. ESBL-producing *Escherichia coli* is an epidemiological risk factor, as these bacteria are mostly associated with human urinary and systemic infections, presenting a therapeutic challenge [1]. This group of bacteria has developed resistance to most β-lactam antibiotics, including penicillins and first-, second-, and third-generation cephalosporins, by producing enzymes that break down these drugs. The core mechanism is the production of hydrolysing enzymes that target the β-lactam ring and open it up, thereby inactivating the antibiotic [2,3].

ESBL enzymes are structurally mutated versions of ancient types of β-lactamases that today are categorised into four different classes (A, B, C, and D) based on the Ambler classification [4]. A typical feature of ESBL enzymes is that they are encoded on mobile genetic elements, typically plasmids [5]. Since, in several cases, these plasmids are conjugative, the spread of ESBL genes, and thus the resistance they carry, can spread effectively among bacteria. This process frequently occurs in the gut flora of humans and animals and has also been observed in other environmental niches [6].

*E. coli* is a common member of the gut flora that can take up ESBL-carrying plasmids, thereby becoming resistant to a broad spectrum of β-lactam antibiotics. International studies have revealed that the presence of ESBL-producing *E. coli* on pig and poultry farms has reached alarming levels [7,8,9,10,11]. Pig farms are considered the greatest source of infection, as farmers can be exposed to these bacteria through direct contact with animals and faecal material. However, a much greater transmission occurs during slaughtering, when ESBL-containing faecal material can contaminate meat and surfaces in the slaughterhouse.

Well-adapted strains can adhere to and survive on both biotic (e.g., meat) and abiotic (e.g., metal, plastic) surfaces [12,13]. Survival is strongly supported by biofilm formation. This extracellular matrix, composed of different polysaccharides and proteins, provides partial or complete protection from environmental effects such as desiccation, UV exposure, disinfectants, and antibiotics [14,15], which makes eliminating them a real challenge.

The role of biofilms in the spread of ESBL-producing *E. coli* has recently been reported in three contexts, reflecting three major target areas for management. According to these reports, ESBL-producing *E. coli* can survive changes in livestock stables and colonise new starter livestock, thereby ensuring the persistence of presenting clones in stables [7,8]. Furthermore, it can colonise different surfaces in the slaughterhouse environment [9], and in the event of faecal contamination, it can be transferred onto the surface of fresh meat, enabling multidrug-resistant bacterial agents to reach human consumers [10]. In humans, ESBL can colonise the gut, and the resulting faecal shedding in the human body serves as a source for bacterial spread in both community and hospital environments and can be the starting point of endogenous infections causing urinary tract, bloodstream, and other infections [11]. Since its first identification in the 1980s, several epidemiological studies have found that most ESBL-producing *E. coli* cases originate from the genitourinary tract [12,13]. A meta-analysis of 62 out of 575 relevant articles found that the global overall prevalence of intestinal carriage of ESBL-producing *E. coli* was 16.5%. The highest carriage rate was found in Southeast Asia (27%), while the lowest overall prevalence was found in Europe (6%). The highest prevalence was observed in Tanzania (76.3%). The research team’s findings suggest that the presence of MDR ESBL-producing *E. coli* is increasing dramatically outside the hospital setting [14].

Due to its importance in survival and spreading, the biofilm is a logical bacterial structure to target. For this reason, it is expected that decontamination methods should not only be antibacterial but also inhibit biofilms and degrade them. Although recent decontamination methods for abiotic and biotic surfaces, such as slaughterhouse equipment and meat, have been demonstrated to be effective, the unpleasant nature of the chemicals used does not meet consumer expectations.

However, the application of certain essential oils (EOs) could solve this problem, since some of them have antibacterial properties and are recognised as generally safe (GRAS) by the U.S. Food and Drug Administration (FDA). Recent studies have widely demonstrated their antimicrobial and antibacterial properties [15,16,17,18].

EOs are mixtures of volatile, aromatic compounds derived from various plant parts, such as leaves, flowers, and fruits. Their chemical composition is highly complex, containing terpenes, terpenoids, phenols, alcohols, aldehydes, and ketones, which exhibit a wide spectrum of biological activities, including antimicrobial, anti-inflammatory, and antioxidant effects [19,20]. The primary advantage of EOs over antibiotics is their broad spectrum of action stemming from their diverse chemical composition, which ranges from general membrane disruption to the inhibition of specific processes, such as nucleic acid and protein biosynthesis or enzymatic functions [21]. Bacterial biofilms represent a major clinical challenge due to their protective extracellular polymeric substance (EPS) matrix, which restricts the penetration of conventional hydrophilic antibiotics. In this context, EOs have emerged as promising anti-biofilm agents. Owing to their lipophilic nature, the hydrophobic constituents of EOs can effectively permeate the EPS matrix and disrupt both the structural integrity of the biofilm and the bacterial cell membranes embedded within it [21,22,23]. Their ability to affect multiple cellular processes, together with their potential to interfere with both the biofilm matrix and embedded bacterial cells, provides a rationale for investigating EOs as a complementary strategy for controlling biofilm-associated infections.

This study aimed to compare the biofilm-forming capacities of 20 different ESBL-producing *E. coli* isolates on polystyrene surfaces with different coatings and on stainless steel. To identify influential factors, this study was conducted using various carrier media and temperatures (14 °C, 23 °C, and 37 °C). The antibacterial and antibiofilm efficacies of 60 different EOs were then tested under various conditions to determine the influence of conditions on biofilm treatment efficacy.

## 2. Results

### 2.1. Drop Plate Method

Results of the drop plate method tests showed that 50 essential oils exhibited antibacterial activity at a concentration of 1%, while 54 EOs were effective at a concentration of 5% at 37 °C. The inhibition zone diameters increased with concentration. Ajowan, bay, cinnamon, citronella, clove, geranium, lime oil (as per Bp), mint, neroli, nutmeg, oregano, palmarosa, petitgrain, and thyme were effective against all 20 ESBL-producing *E. coli* isolates. At a concentration of 5% EO, the list expanded to include cajeput, clary sage, juniper, melissa, orange, peppermint, tea tree, rose geranium, sage, and ylang ylang EOs. Of the isolates, NNK1 was the most resistant in the presence of a 1% EO concentration, whereas JM23 proved to be the most resistant at 5% EO concentration.

**Figure 1:**
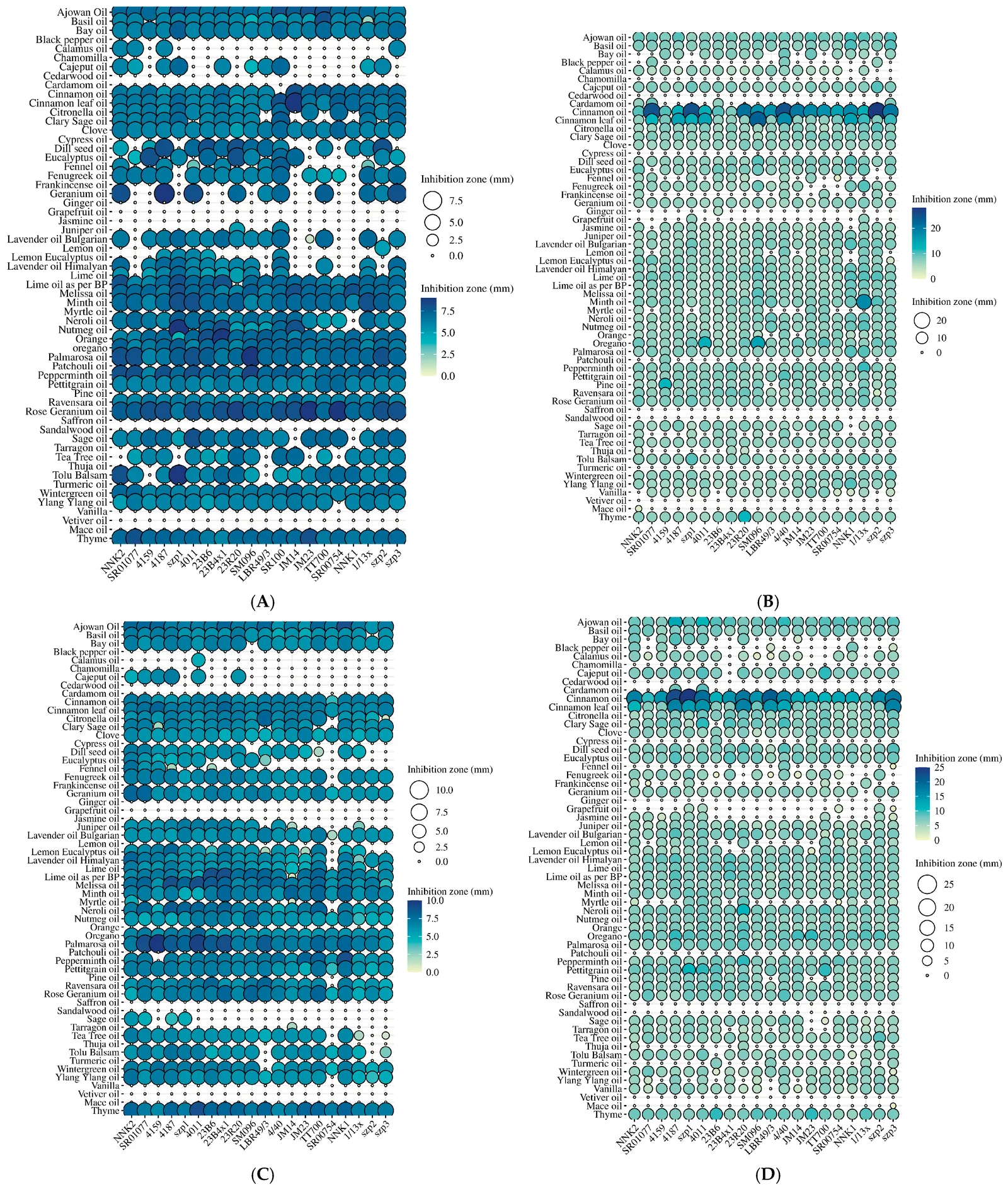

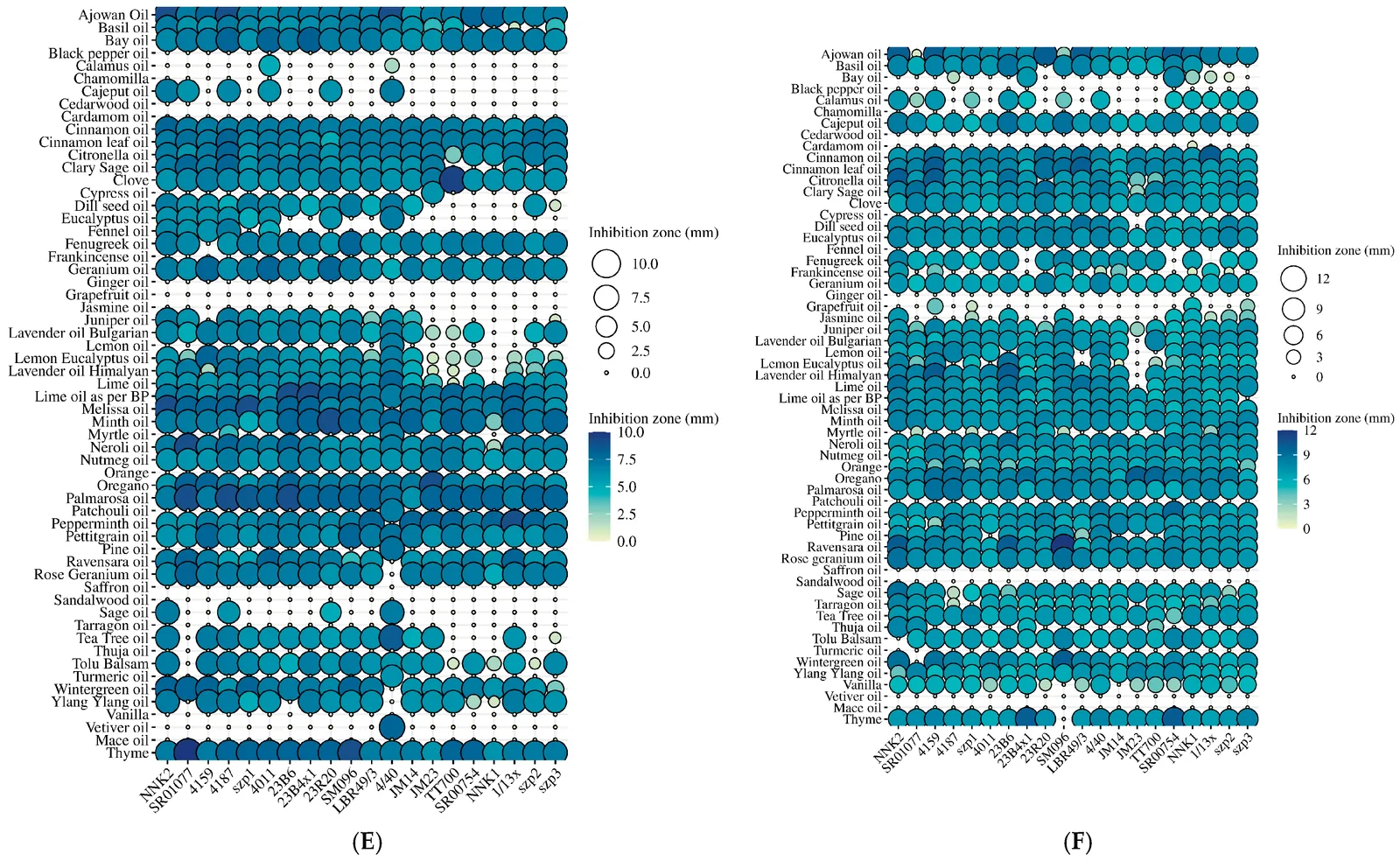
Antibacterial activities of 60 essential oils on the 20 tested ESBL-producing *E. coli* isolates, tested with drop plate method at 14 °C (**A**,**B**), 23 °C (**C**,**D**), and 37 °C (**E**,**F**), and by using 1% (**A**,**C**,**E**) and 5% (**B**,**D**,**F**) EOs. The inhibition zone sizes are presented in different colours and balloon sizes in the SRPlot balloon plot. Results of the drop plate method tests showed that 50 Eos exhibited antibacterial activity at a concentration of 1%, while 54 EOs were effective at a concentration of 5% at 37 °C. The inhibition zone diameters increased with concentration. Ajowan, bay, cinnamon, citronella, clove, geranium, lime oil (as per Bp), mint, neroli, nutmeg, oregano, palmarosa, petitgrain, and thyme were effective against all the 20 ESBL-producing *E. coli* isolates.

At 14 °C, rose geranium oil was the most effective at 1% EOs, with an inhibition zone of 6.5–9 mm. At 23 °C, palmarosa was the most effective, with an inhibition zone of 6–10 mm. At 37 °C, ajowan EO had an inhibition zone of between 6.5 and 9 mm, while thyme had an inhibition zone of between 6 and 10 mm. Cinnamon oil was the most effective at both 14 °C and 23 °C in the case of 5% EO. The inhibition zone was between 10 and 28 mm at 14 °C and between 10 and 25 mm at 23 °C. At 37 °C, the inhibition zone was between 6 and 10.1 mm. While 5% EOs were more effective at the temperatures of 14 °C and 23 °C, no such notable difference was revealed at 37 °C between the applied EO concentrations (Figure 1).

### 2.2. Biofilm Formation

In the statistical analysis of overall biofilm formation, first, the Levene test was used to assess the homogeneity of variances. The *p*-value (*p* < 0.001) here led us to reject the null hypothesis. The Shapiro–Wilk test revealed that our data did not follow a normal distribution for every single strain (*p* < 0.001), so we proceeded with the Kruskal–Wallis test.

Significant differences were found among the strains, culture media, and temperatures (*p* < 0.001), but not among the plates used (*p* = 0.525).

**Figure 2:**
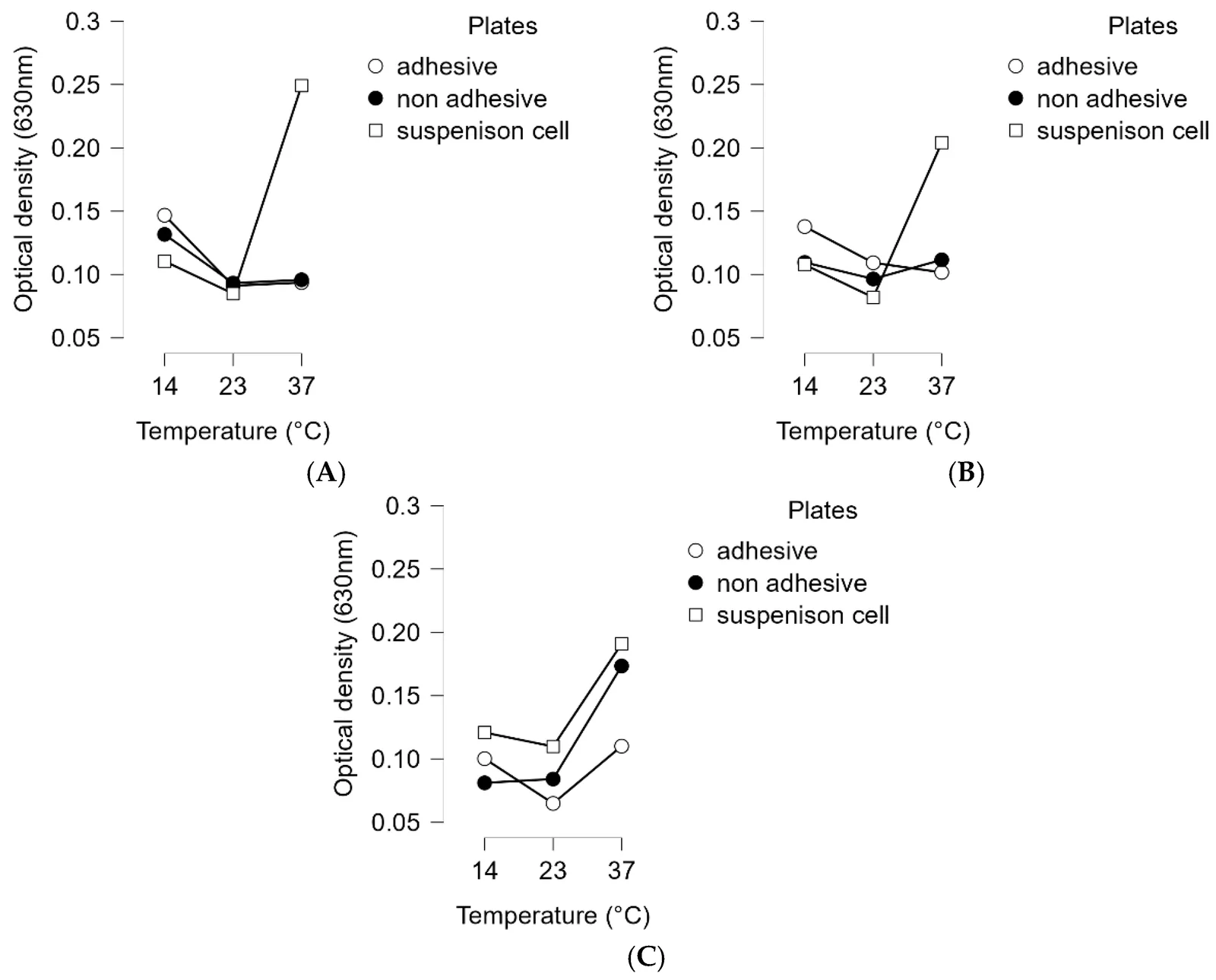
The influential effects of the quality of the carrier medium (**A**–**C**), the plates, and the temperature on the biofilm formation of the 20 ESBL-producing *E. coli* isolates. The graph shows the cumulative data for (**A**) LB, (**B**) MH-II, and BHI (**C**) of the results from the 96-well plates. In all plate types, it is clearly evident that biofilm formation was most intense at 37 °C.

The biofilm formation of the 20 different ESBL-producing *E. coli* investigated was influenced by all three investigated factors: surface quality, carrier medium, and temperature (Figure 2). In all three-culture media (Figure 2A–C) and for almost all plate types, it is clearly evident that biofilm formation was most intense at 37 °C.

As the temperature rises from 14 °C to 23 °C, the amount of biofilm stagnates or decreases slightly; then, as the temperature rises from 23 °C to 37 °C, it began to increase dramatically (especially on the adhesive and suspension plates).

Examining the plates, we observed that the suspension and adhesive plates exhibited the highest optical density (0.1–0.25).

On the non-adhesive plate, low biofilm values were consistently observed at all three tested temperatures. This plate hinders the physical attachment of the biofilm.

### 2.3. Isolate 23R20 as the Best Biofilm Former Among the Investigated ESBL-Producing E. coli Isolates

**Figure 3:**
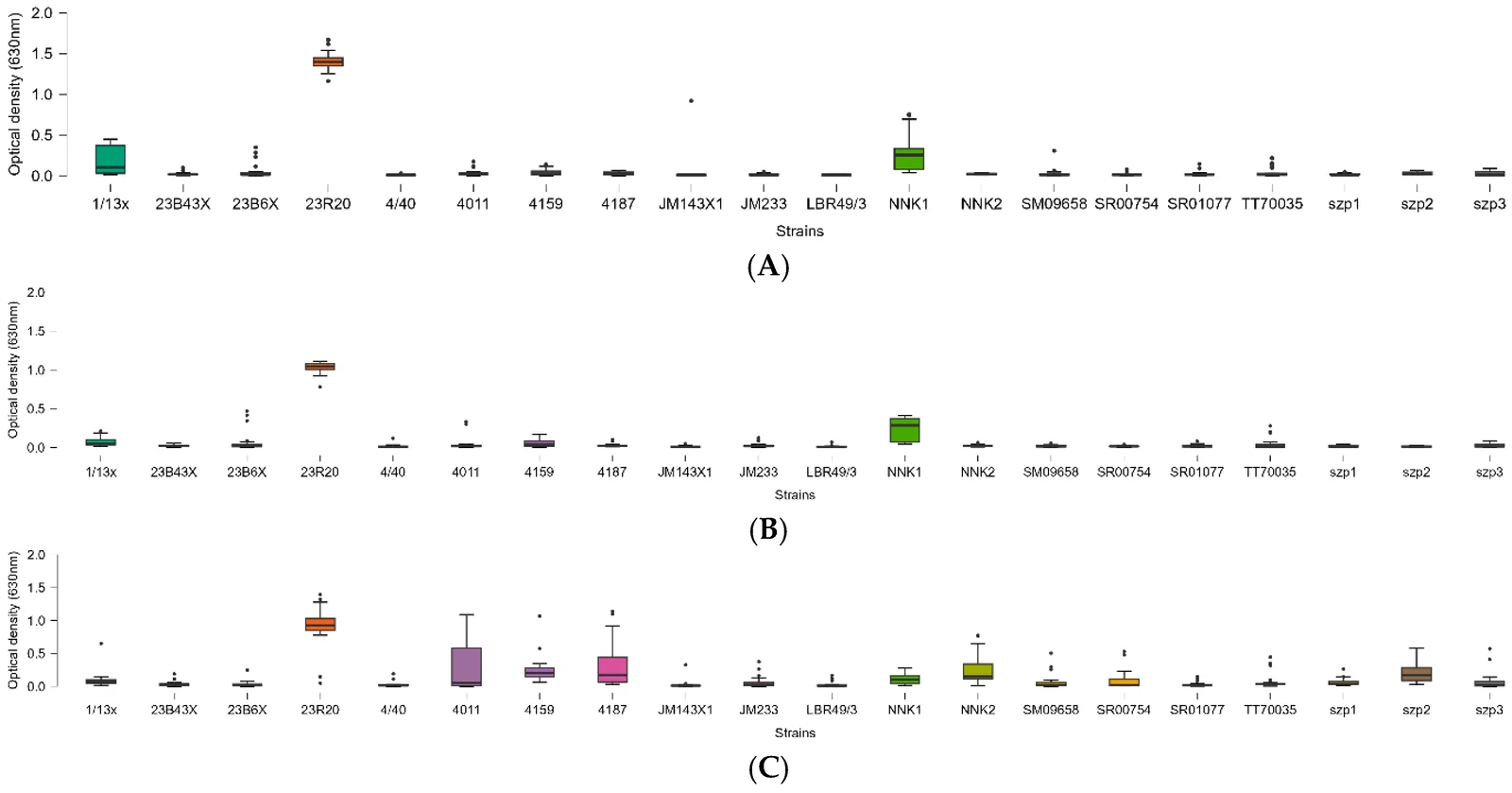
Biofilm-forming abilities of the tested 20 ESBL-producing *E. coli* isolates at 14 °C (**A**), 23 °C (**B**), and 37 °C (**C**) are shown. This is a cumulative graph that takes into account the results for all three nutritional solutions. At 14, 23, and 37 °C, the ESBL-producing *E. coli* isolate 23R20 showed the strongest biofilm formation. At 14 °C, 1/13x (OD: 0.017–0.449) and NNK1 (0.039–0.759) also exhibited good biofilm formation, and at 23 °C, 1/13x formed the weakest biofilm of these three isolates, with an optical density of 0.016–0.212.

For further experiments, the ESBL-producing *E. coli* isolate that formed the firmest biofilms was selected. At 14, 23, and 37 °C, the ESBL-producing *E. coli* isolate 23R20 showed the strongest biofilm forming formation. At 14 °C, 1/13x (OD: 0.017–0.449) and NNK1 (0.039–0.759) also exhibited good biofilm formation (Figure 3A). At 23 °C, 1/13x formed the weakest biofilm of these three isolates with an optical density of 0.016–0.212 (Figure 3B). NNK1 formed a matrix with an optical density of 0.048–0.412, while 23R20 formed a matrix with an optical density of 0.126–1.114. Thirty-seven degrees significantly promoted biofilm formation in all isolates, but under this condition, the optical density of the formed and stained extracellular matrix of isolate 23R20 ranged from 0.05 to 1.395. This value was characteristically higher than those of 1/13x and NNK1, which ranged from 0 to 0.654 and 0.017 to 0.528, respectively. The graph shows several temperature-dependent isolates.

NNK1 formed a moderate amount of biofilm at 14 °C (A) and 23 °C (B), but surprisingly, its biofilm production decreased at 37 °C (C). For this strain, cooler ambient temperatures favour the formation of the biofilm matrix. Looking at graph C (37 °C), several isolates (e.g., 4011, 4159, 4187, and szp2) are seen to be completely inactive at 14 °C and 23 °C but began to produce a visible amount of biofilm at 37 °C.

The statistical analysis also revealed significant differences between the results of the tested isolates in terms of culture media and temperatures (*p* < 0.001). Based on the Kruskal–Wallis test, no significant differences were found between the plates used (*p* = 0.854).

Based on Stepanovic’s biofilm formation groups, we examined the biofilm-forming capacity of *E. coli* isolates in 27 different combinations of three types of media, three temperatures, and three types of plates.

The 23R20 isolate is a constitutive biofilm former. Out of the 27 possible combinations, it formed a strong biofilm 26 times and a moderate biofilm once. It never failed to form a biofilm under any circumstances. This suggests that its biofilm formation is independent of external factors such as temperature, culture medium, and surface. From a biological perspective, this indicates an extremely resistant strain.

NNK1 is a fundamentally good biofilm-forming isolate with the second-best properties, but its performance depended on the environment. Out of 27 combinations, NNK1 formed a weaker biofilm on 12 occasions. However, it never failed to form a biofilm under any circumstances. The 1/13x and 4187 isolates consistently form biofilms, most frequently in the weak category (Table 1.).

Finally, environment-sensitive isolates belong in the next category. Although these are capable of forming strong or moderate biofilms, they remain completely inactive under many conditions (e.g., in the 8–13 combination). One example is isolate 4159, which exhibits moderate biofilm formation an exceptionally high number of times (10 times), suggesting that it consistently responds to certain environmental stimuli in a moderate way. Finally, there are weak or biofilm-negative strains, such as 23B43X, SR01077, 4/40, and LBR49/3.

### 2.4. Scanning Electron Microscope Analysis

**Figure 4:**
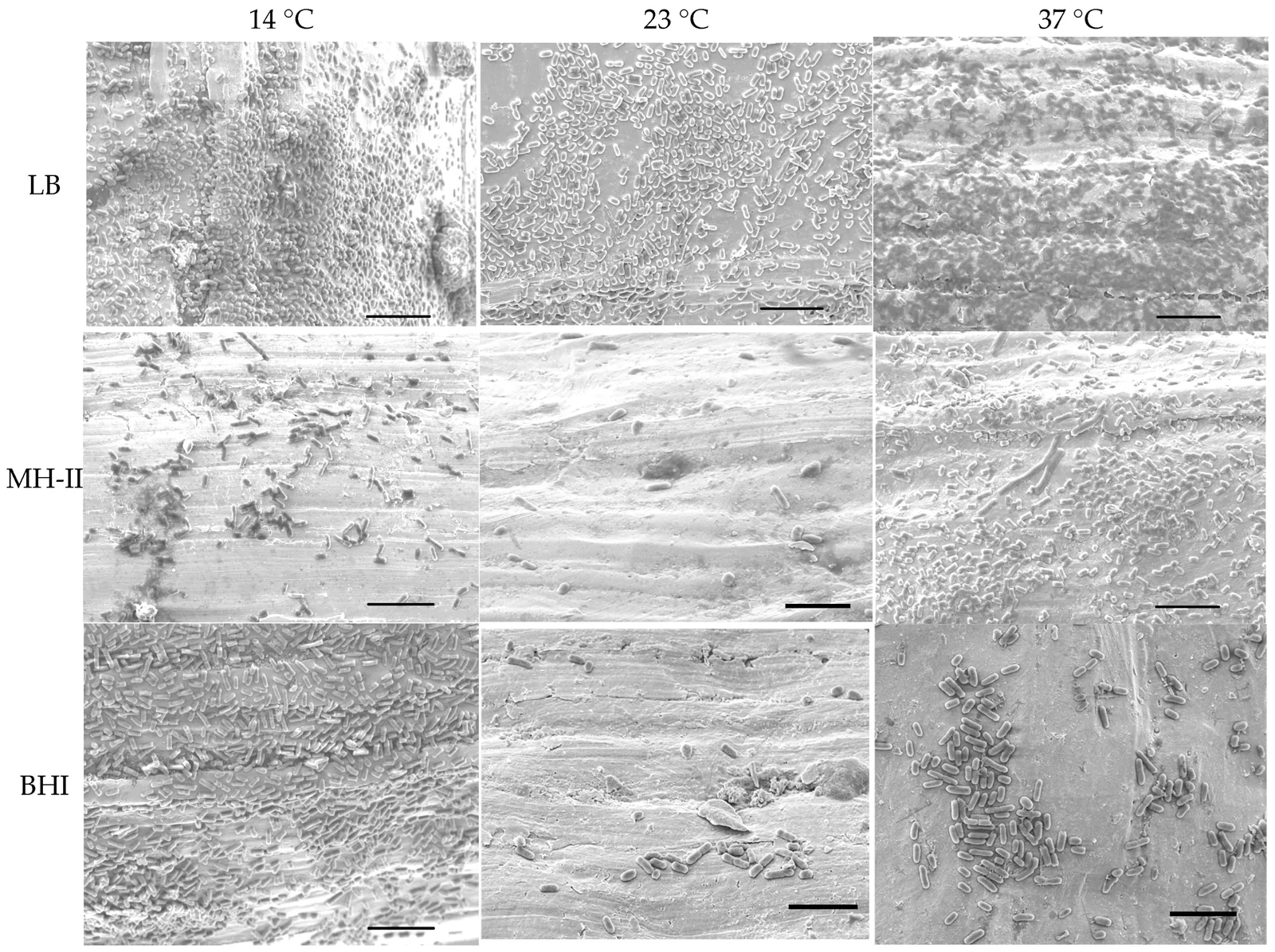
The biofilm formation capacity of the 23R20 ESBL-producing *E. coli* isolate on stainless steel surface at temperatures 14 °C, 23 °C, and 37 °C, in LB, MH, and BHI media (scale: 10 µm). Scanning electron microscopy of the 23R20 ESBL *E. coli* isolate reveals intact, smooth-surfaced, rod-shaped cells. Their distribution on the stainless steel surface is variable.

Scanning electron microscope analysis was used to evaluate the ability of the ESBL-producing *E. coli* isolate 23R20 to form biofilms on a stainless-steel surface. The cells exhibited their characteristic rod shape with smooth surfaces and were free from deformation, collapse, or wrinkling. Depending on the visualised microscopic field, the cells were scattered and loosely adhered to the steel surface. Groups of adhered bacteria were clearly visible, but no distinct classical biofilm formation could be observed (Figure 4).

### 2.5. Biofilm Inhibition

**Figure 5:**
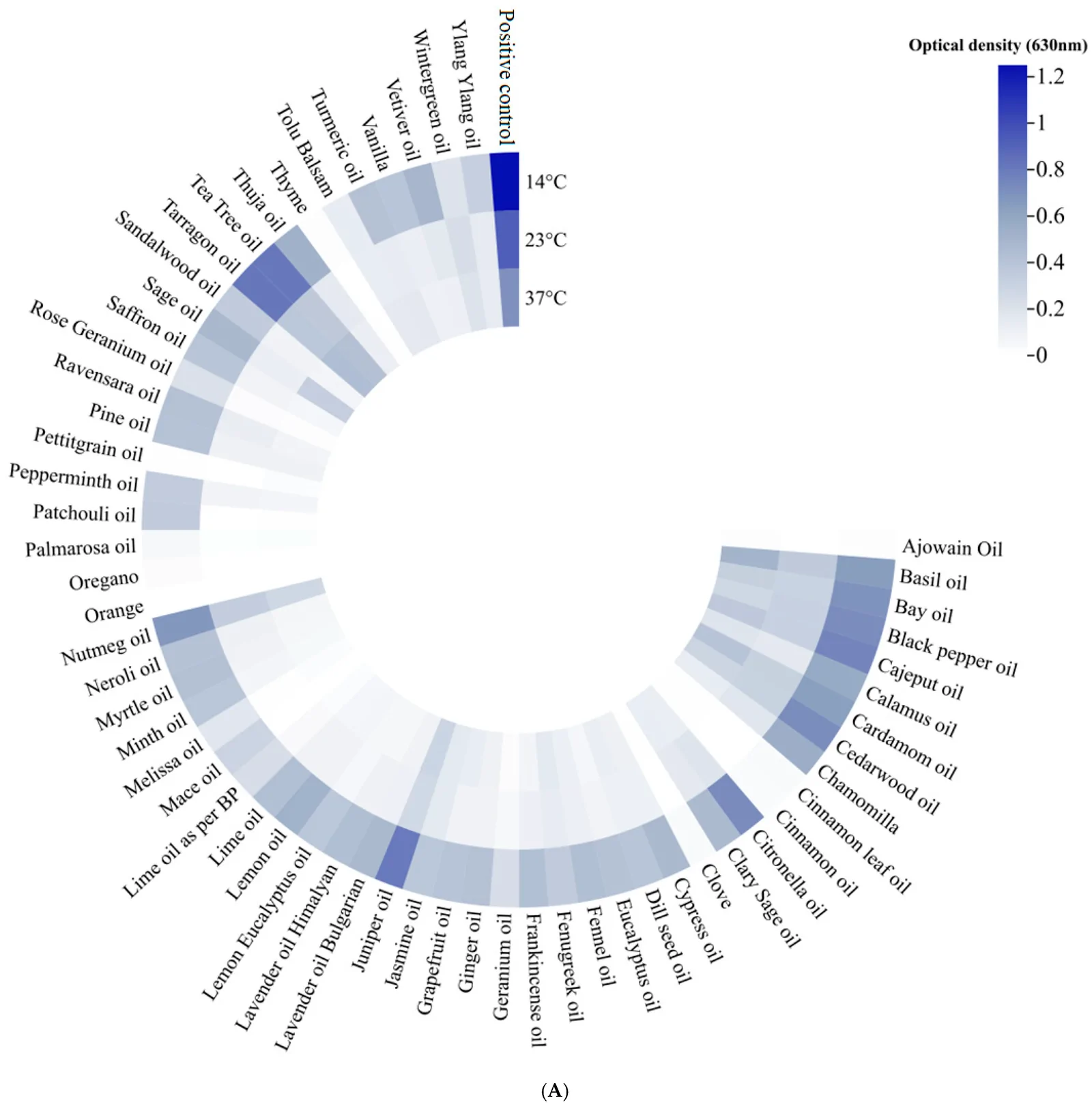

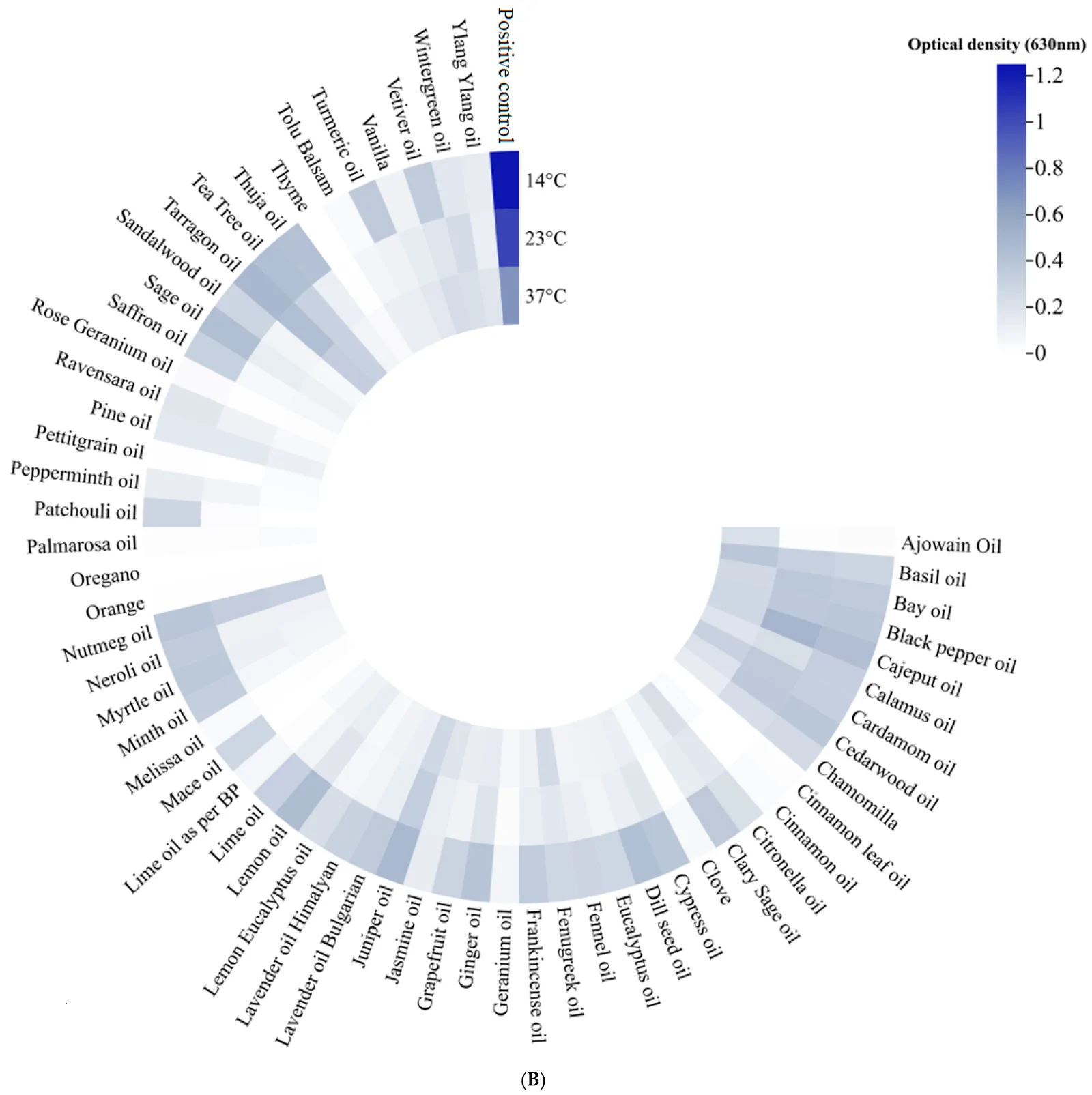
Cumulative diagram of the biofilm-inhibitory potential of 60 essential oils on the ESBL-producing *E. coli* isolate 23R20. Bacterial cells were grown in MH-II (**A**) and BHI (**B**) in the presence of 0.1% EOs, at 14 °C for 48 h, and at 23 °C and 37 °C for 24 h. The formed biofilm matrices were stained, resolubilised, and evaluated by measuring their optical density values, as shown in the heatmap (https://www.chiplot.online/ accessed on 4 March 2026). Optical densities are represented by colours. The darker the colour, the higher the OD value at 630. Cinnamon, clove, palmarosa, petitgrain, oregano, and thyme oils were the strongest candidates for anti-biofilm activity, as these EOs showed similar effects at most temperatures and reduced the optical density to between 0.003 and 0.015, regardless of the nutrient medium.

The ESBL-producing *E. coli* strain 23R20 was used in the experiments to test inhibitory effects on biofilms. Without exposure to any essential oils, the mass of the biofilm formed was greater (shown as a darker colour in Figure 5) in both MH-II and BHI. Compared to these control values, the drastically reduced mass of the biofilms formed in the presence of 0.1% EOs was evident from the decreased OD_630_ values. Some temperature dependence was observed in the OD values, with 14 °C proving to be the temperature at which matrix formation was most supported.

The EOs exhibited various inhibitory effects on biofilm formation. However, clove, oregano, thyme, petitgrain, palmarosa, orange, and cinnamon demonstrated the most pronounced inhibitory effect, with no biofilm formation observed when these EOs were applied to MH-II. A similar pattern was observed in the case of BHI, albeit with less activity; however, the inhibition was not complete. It can generally be stated that the OD values obtained were lower in the presence of BHI than for the biofilms formed in MH-II. This was particularly evident in experiments conducted at 14 °C.

Regardless of the carrier medium used, weaker inhibition was observed with basil (OD_630_: 0.285–0.64), bay (OD_630_: 0.26–0.699), black pepper (OD_630_: 0.27–0.72), cardamom (OD_630_: 0.31–0.64), and cedarwood (OD_630_: 0.20–0.72), while neroli (OD_630_: 0.063–0.41), orange (OD_630_: 0.001–0.0115), and lemon (OD_630_: 0.068–0.52) had a moderate effect (Figure 5).

The least effective EOs were tarragon, tea tree, basil, bay, and black pepper. However, biofilm formation was observed in the presence of these oils, with the respective OD_630_ values reaching 0.378–0.82, 0.30–0.82, 0.285–0.64, 0.26–0.699, and 0.27–0.72. Temperature-dependent activity was observed in patchouli, juniper, peppermint, thuja, saffron, and sandalwood. Cinnamon, clove, palmarosa, petitgrain, oregano, and thyme were the strongest candidates for anti-biofilm activity, as these EOs showed similar effects at most temperatures and reduced the optical density to between 0.003 and 0.015, regardless of the nutrient medium.

To complement the biofilm biomass measurements, the effect of EO co-incubation on bacterial viability was assessed by enumeration of colony-forming units (CFU). The detection limit of the assay was 100 CFU/mL. The viable bacterial counts varied considerably depending on the EO, incubation temperature, and culture medium. At 14 °C, ajowan, cinnamon-bark, cinnamon leaf, clove, orange, oregano, palmarosa, petitgrain, and thyme resulted in viable counts below the detection limit (<100 CFU/mL) in both MH-II and BHI. At 23 °C, viable cells were detected for several EOs, most notably palmarosa (55,000 ± 7071.1 CFU/mL in MH-II and 27,500 ± 3535.5 CFU/mL in BHI), petitgrain (12,500 ± 3535.5 CFU/mL in MH-II and <100 CFU/mL in BHI), cinnamon bark (900 ± 1272.8 CFU/mL in BHI), and ajowan (<100 CFU/mL in MH-II). At 37 °C, viable cell counts for the EOs were below the detection limit (<100 CFU/mL) in both media. In comparison, the positive control showed substantially higher viable bacterial counts, ranging from 3.6 × 10^8^ to 8.2 × 10^8^ CFU/mL across the tested conditions. These findings demonstrate that EO exposure during biofilm development substantially reduced viable bacterial recovery compared with the untreated control, although the magnitude of the effect varied among EOs, temperatures, and culture media.

Statistical analysis of the results revealed significant differences (*p* < 0.001) in the ability of the EOs and temperatures to inhibit biofilm formation. Therefore, inhibiting ESBL-producing *E. coli* biofilm formation is influenced by the type of EO (df:59) and the temperature (df:2) used. Nutrient solution (df:1) did not affect biofilm formation based on the Kruskal–Wallis test (*p* = 0.059).

### 2.6. Biofilm Removal

**Figure 6:**
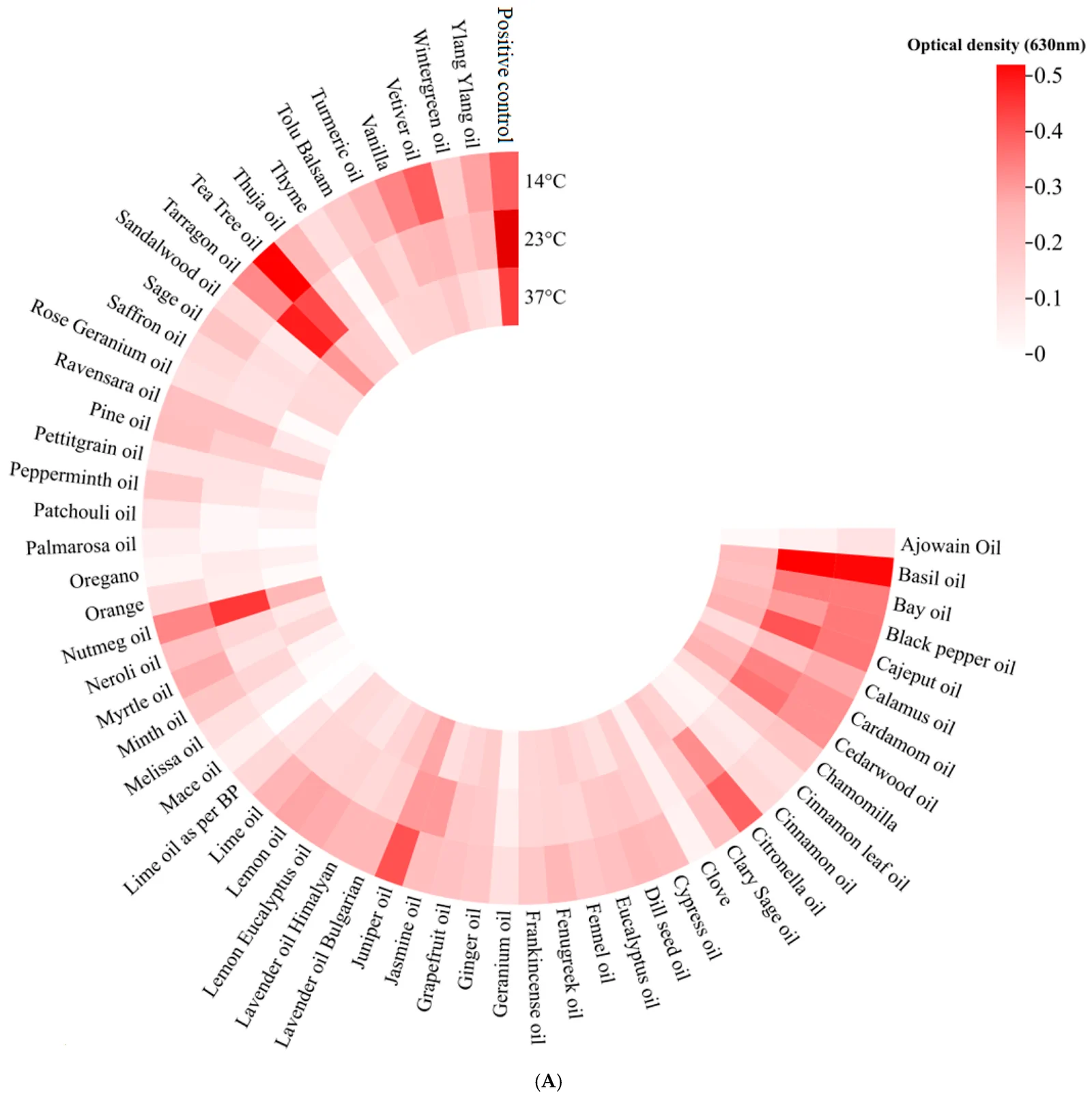

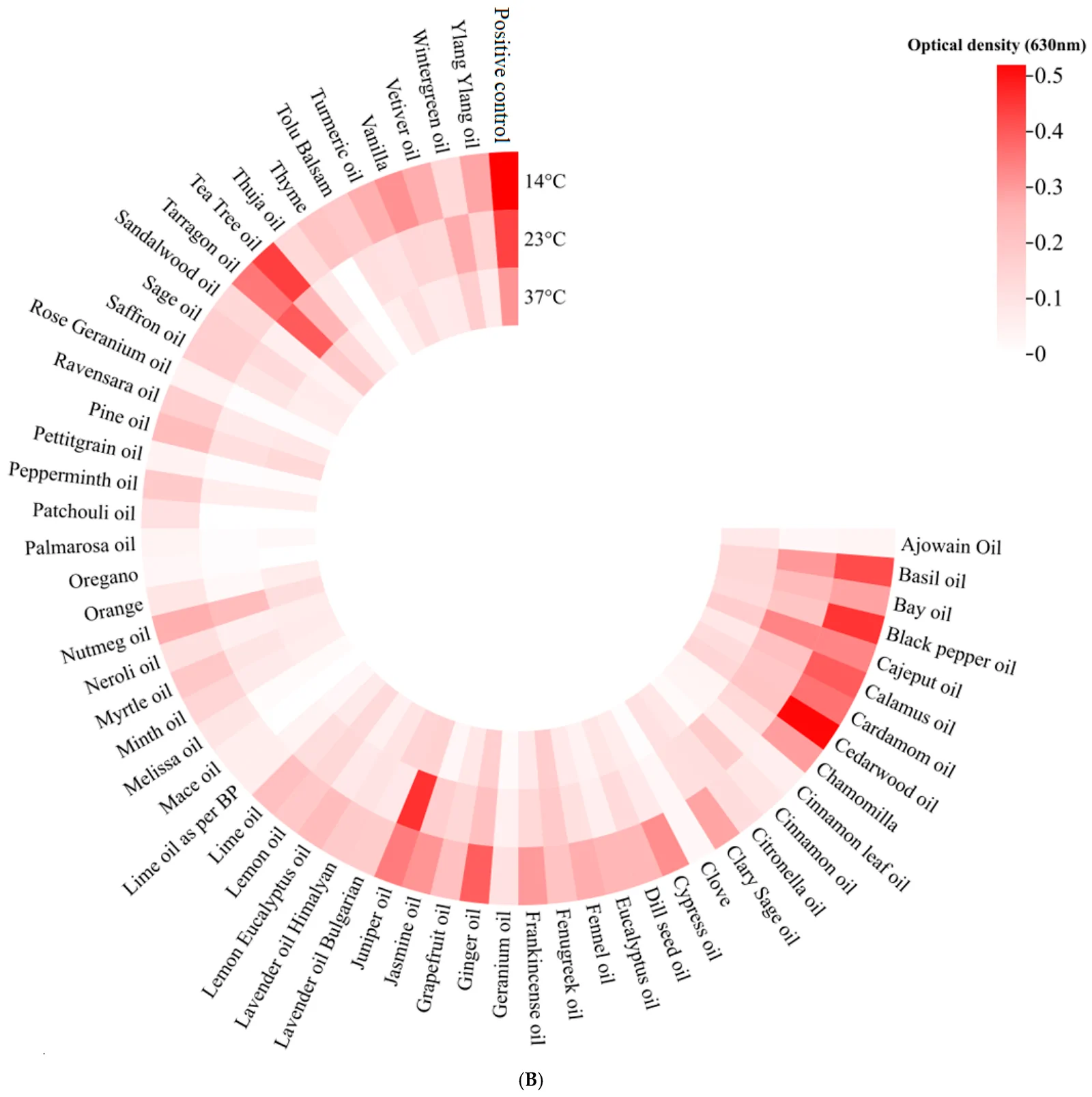
Comparison of the biofilm-disruptive effects of the 60 essential oils applied in (**A**) MH-II and (**B**) BHI media at 0.1% end concentrations at temperatures of 14 °C, 23 °C, and 37 °C. The mature biofilm of the ESBL-producing *E. coli* isolate 23R20 was used for the experiments. The most effective EOs were thyme, oregano, clove, and cinnamon, as these EOs showed similar effects at most temperatures, by lowering the optical densities to 0.0035–0.2, 0.01–0.073, 0.0095–0.059, and 0.034–0.113, respectively. In the graph, darker fields indicate thicker biofilms and therefore a lower disruptive effect (https://www.chiplot.online/circle_heatmap.html, accessed on 6 June 2026).

These experiments aimed to degrade mature biofilms of the ESBL-producing *E. coli* isolate 23R20 using essential oils. The final EO concentration applied was 0.1%, and the experiments were performed at 14 °C, 23 °C, and 37 °C. The disruptive effects were tested in MH-II (Figure 6A) and BHI (Figure 6B) culture media to determine the influence of the carrier medium on the efficacy of the EOs. The white/pale pink segments of the graphs (Figure 6) indicate low optical densities, i.e., the strong biofilm-degrading activity of the EOs. Clearly, optical densities were generally lower when the EOs acted in MH-II than in BHI.

The most effective EOs were thyme, oregano, clove, and cinnamon, as these EOs showed similar effects at most temperatures, lowering the optical densities to 0.0035–0.2, 0.01–0.073, 0.0095–0.059, and 0.034–0.113, respectively. This demonstrates their strong ability to degrade mature biofilms. In contrast, certain citrus derivatives (e.g., orange, lavender, geranium, and pine) and some menthol-type oils, such as peppermint, showed a weak biofilm-disrupting effect and did not significantly destroy mature biofilms.

To determine the bactericidal efficacy of 0.1% (*v*/*v*) EOs, the number of viable cells against pre-formed biofilms was determined. The detection limit of the assay was 100 CFU/mL. Ajowan, cinnamon leaf, and clove showed strong bactericidal efficacy, with viable cell counts falling below the detection limit (<100 CFU/mL) at all temperatures and media evaluated. For example, orange oil showed residual viable cells at 14 °C, with 450 ± 70.7 CFU/mL in MH-II and <100 CFU/mL in BHI, whereas at 23 °C and 37 °C, viable cell counts were below the detection limit (<100 CFU/mL) in both media.

Oregano oil showed similarly strong bactericidal activity, with viable cells detected only at the detection limit (100 CFU/mL) in BHI at 14 °C. For the remaining EOs, the bactericidal activity varied depending on the incubation temperature and culture medium. Overall, higher incubation temperatures were associated with lower viable cell counts. At 37 °C, ajowan, cinnamon bark, cinnamon leaf, clove, orange, oregano, palmarosa, and thyme reduced the viable cell count below the detection limit in both tested media, while petitgrain yielded a minimal residual population near the detection limit (<100 CFU/mL) in BHI. The positive control showed consistently high viable cell counts under all tested conditions, ranging from 1.8 × 10^7^ to 4.0 × 10^7^ CFU/mL, confirming the presence and recovery of viable bacteria in the experimental system.

In most cases, the trend was clear in the mean that the lower the temperature was, the lower the EO’s biofilm disruptive effect was. However, there were discrepancies in the mean, showing that at 23 °C, the antibiofilm effect was less compared to the other two tested temperatures. This was the case with tarragon, nutmeg, jasmine, cajeput, and cedarwood oils. In general, EOs were most effective at higher temperatures, leaving less biofilm on surfaces.

Kruskal–Wallis test were used to analyse how the optical densities after the biofilm degradation by different EOs were influenced by temperature (df:2), medium (df:1), and EO (df:59). The nonparametric analysis confirmed significant differences (*p* < 0.001) between the EOs, temperatures, and culture media used.

### 2.7. Effect of Ajowan, Clove, Lime, Palmarosa, Oregano, and Thyme Oils on Bacterial Morphology

**Figure 7:**
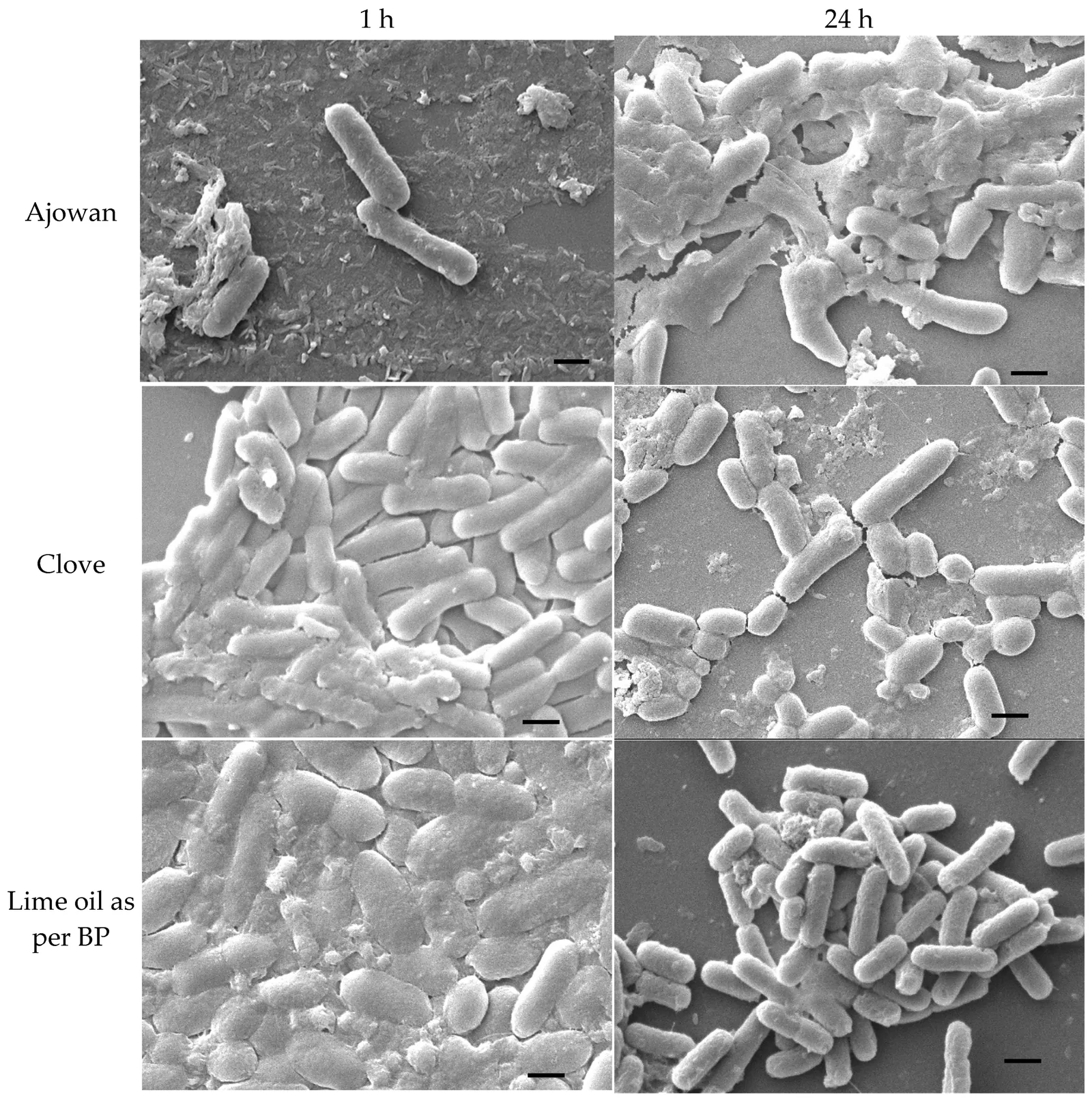

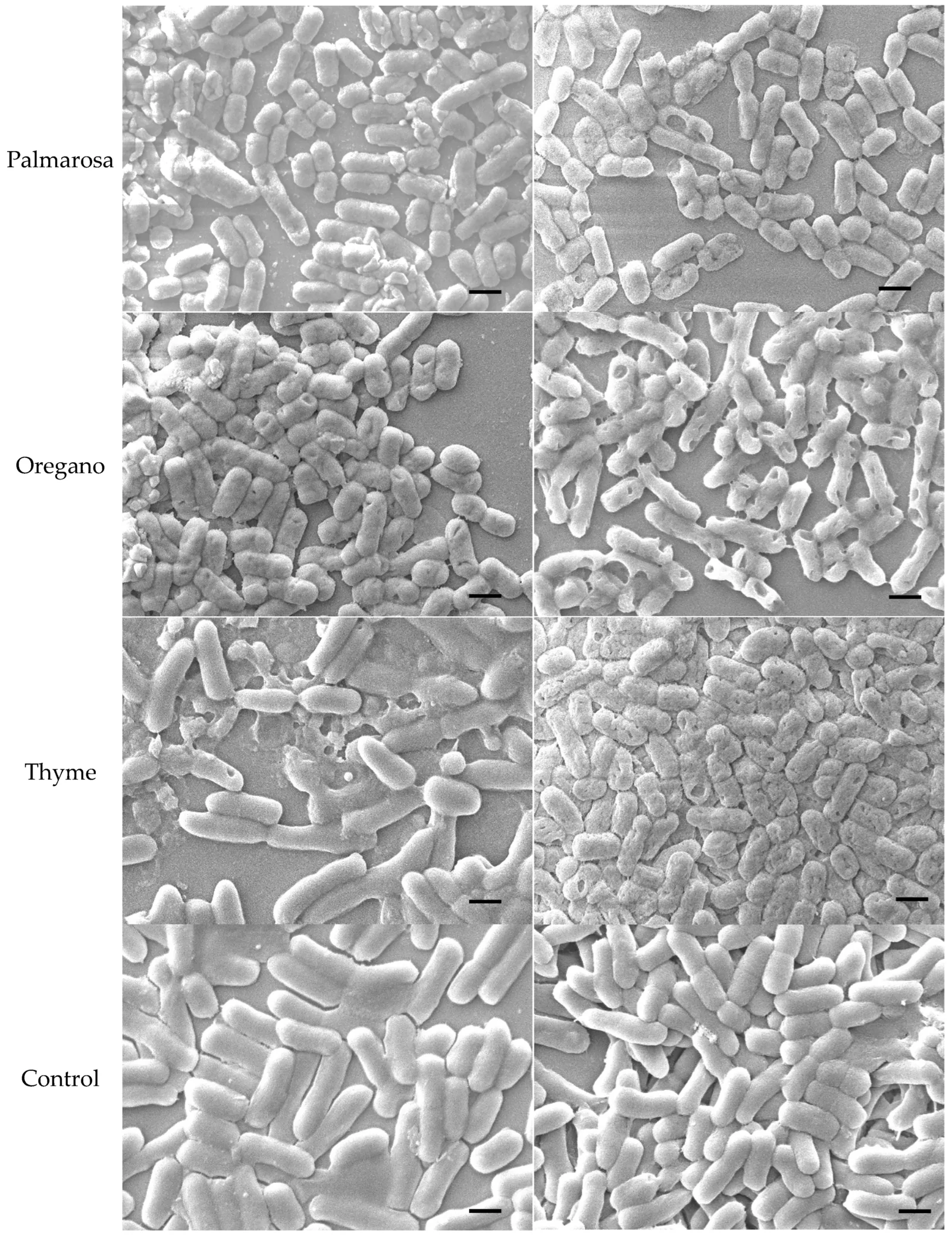
Influential effects of ajowan, clove, lime, palmarosa, oregano, and thyme oils on the morphology of the ESBL-producing *E. coli* isolate 23R20. The cells were incubated in liquid cultures for 1 h and 24 h, after which they were centrifuged and prepared for SEM analysis. Ajowan, clove, lime, palmarosa, oregano, and thyme all caused morphological damage; however, the incubation period played a significant role in the effect on morphology for certain essential oils. The most striking change was observed with thyme. The scale bar sizes are: 1 μm.

The effect of six different essential oils on the morphology of the ESBL-producing *E. coli* isolate 23R20 was tested. Ajowan, clove, lime oil (as per BP), palmarosa, oregano, and thyme EOs were co-incubated with the bacterial isolate for 1 and 24 h, as previously described [24,25]. Scanning electron microscopy (SEM) images of the untreated cells showed that the ESBL-producing *E. coli* cells formed dense aggregates with clearly defined individual cells (Figure 7). Following treatment, the bacteria became elongated when exposed to ajowan oil for 1 h. After 24 h, the cells became destabilised, with amorphous cell structures, distorted shapes, and irregular contours. Clove oil did not result in drastic morphological changes after 1 h of incubation; however, after 24 h, the cells had become spectacularly shortened, and some had become elongated. However, only a few cells exhibited the typical morphology of *E. coli* (see the control sample for comparison). Regarding lime oil, the changes appeared more drastic after one hour; after 24 h, however, the cells had regained their morphological fitness, becoming more similar to the control. In the case of oregano, palmarosa, and thyme, after 24 h, the bacterial cells exhibited different morphologies and several ghost cells, indicating punctured bacteria.

The values (e.g., 83.32% carvacrol and 8.33% thymol) represent the actual final treatment concentrations applied in the assay. These concentrations were selected based on the major active components identified by GC-MS analysis. If we examine only the carvacrol and thymol components present in oregano and thyme, we see that after 1 h of incubation, cells with cytoplasm leaking out and indentations on the surface of some cells can be seen after treatment with 8.33% thymol (oregano) and 8.89% carvacrol (thyme). When using these concentrations, the incubation time did not significantly affect the effectiveness of the components.

With 64.76% thymol (thyme), it can be seen that the cells underwent significant morphological changes with increasing incubation time. In the first hour, the shape of the cells was still rod/coccoid, and then after 24 h, the cells flattened out and took on an amorphous shape.

With 83.32% carvacrol (oregano), pores can be seen on the surface of the bacteria in the first hour, and after 24 h of incubation, the shape of the cells is more amorphous (they have lost turgor).

**Figure 8:**
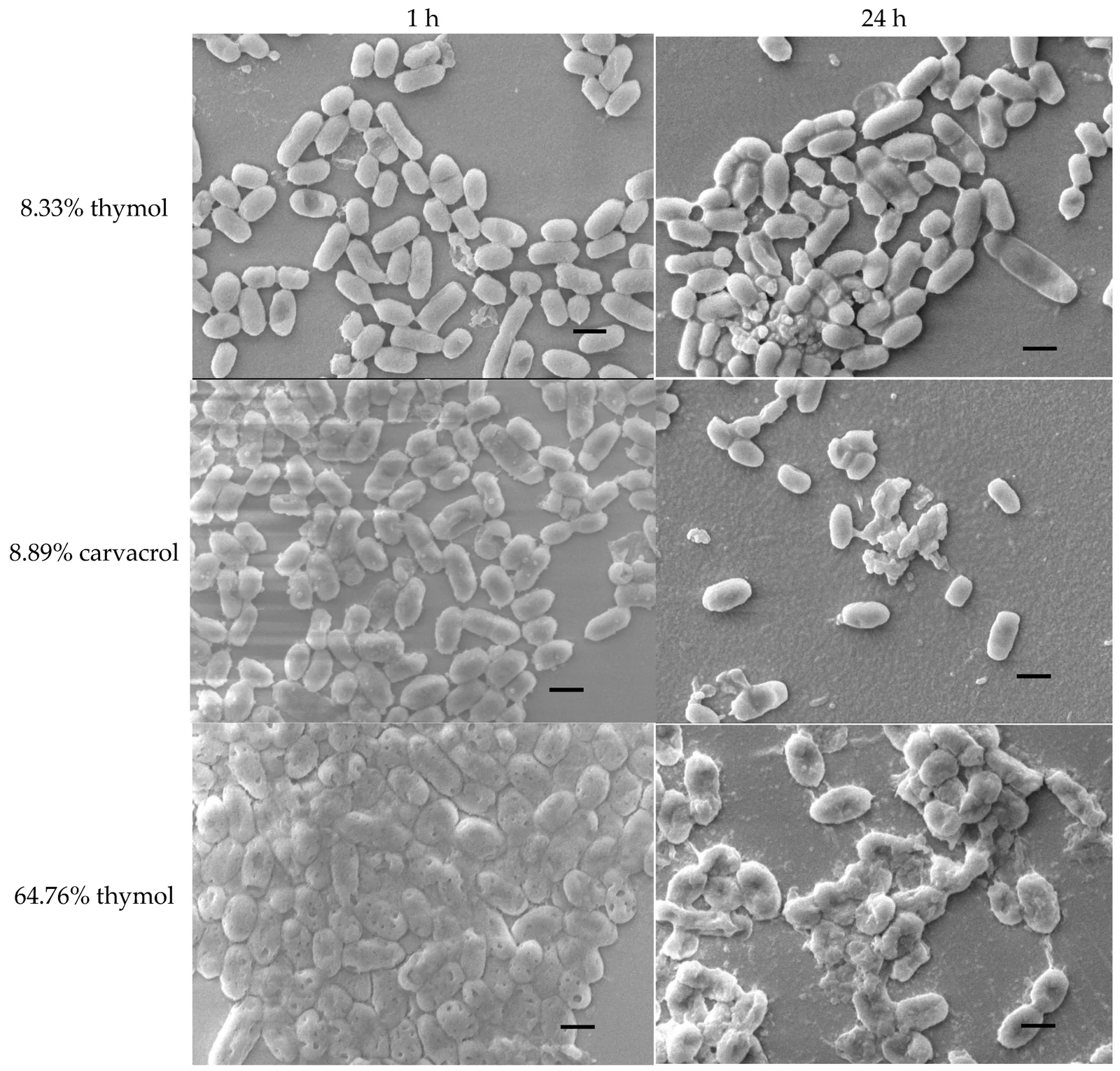

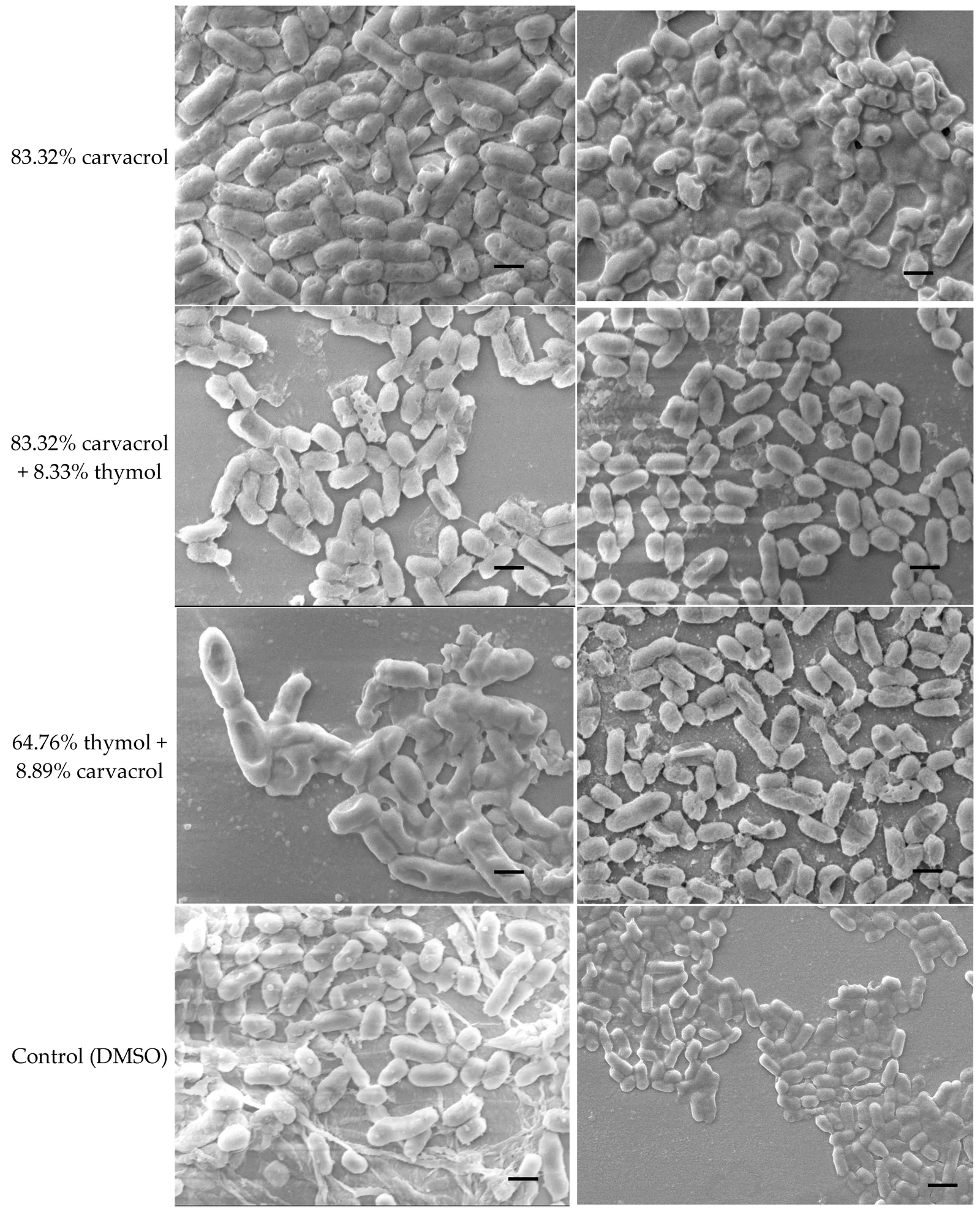
Effect of carvacrol (8.89% and 83.33%) and thymol (8.33 and 64.7%) components and their combination on isolate 23R20. Cells were incubated in liquid cultures for 1 and 24 h, and then centrifuged and prepared for SEM analysis. Thymol and carvacrol (low/moderate concentrations) cause surface depressions and cytoplasmic leakage within one hour; their efficacy remains stable over time. At high concentrations (64.76% thymol/83.32% carvacrol), they cause progressive damage over 24 h, leading to flattened, amorphous cell shapes, the formation of surface pores, and a loss of turgor. In the case of the mixtures, significant cell-disrupting effects are visible in the images after just one hour of incubation, and this effect persists. The DMSO control did not cause any morphological changes. Scale bars: 1 μm.

If we prepare a cocktail of components with concentrations corresponding to the carvacrol (83.32%) and thymol (8.33%) in oregano, and incubate the bacteria in it for 1 and 24 h, we see that significant damage can be observed on the membrane in the first hour. With increasing incubation time, numerous “ghost” cells (cells that have lost their internal content) appear in the background. In the case of a mixture of 64.76% thymol and 8.89% carvacrol, damage to the morphology is already visible after 1 h, which increases further after one day of incubation (Figure 8).

### 2.8. Bioautographic Analyses

**Figure 9:**
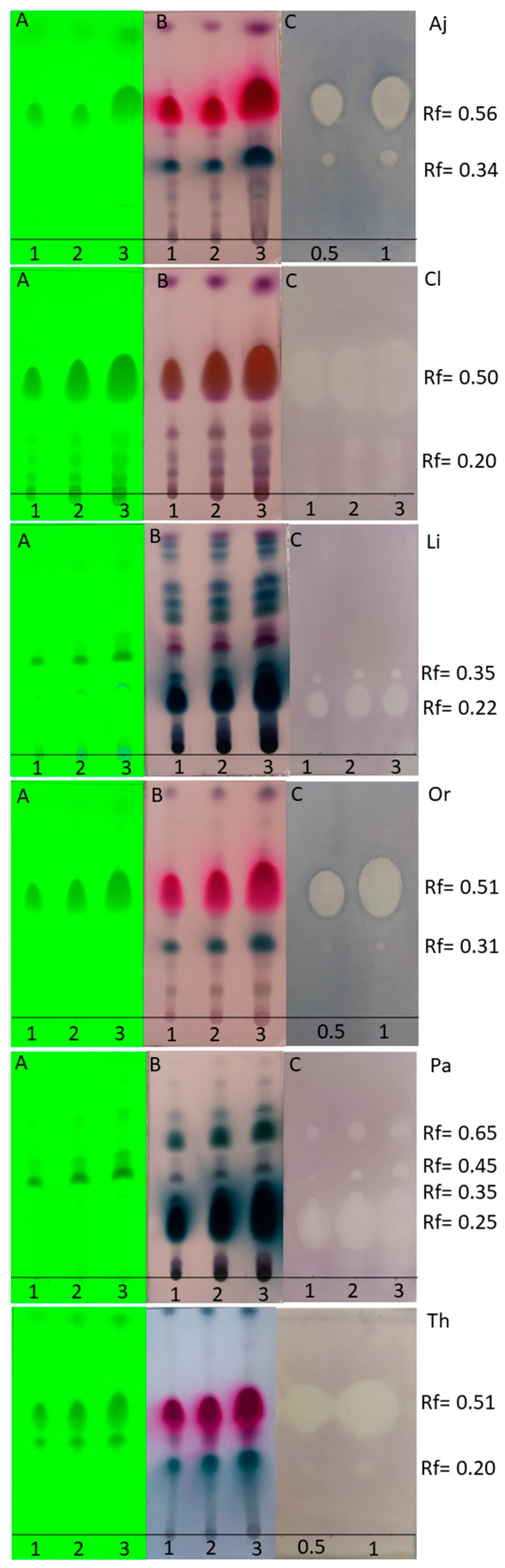
Thin-layer chromatographic separation and direct bioautography were performed on *Trachyspermum ammi* (Aj, ajowan), *Syzygium aromaticum* (Cl, clove), *Citrus aurantifolia* (Li, lime), *Origanum vulgare* (Or, oregano), *Cymbopogon martinii* (Pa, palmarosa), and *Thymus vulgaris* (Th, thyme) against ESBL-producing *Escherichia coli*; these were performed on silica gel 60 F254 TLC plates using a toluene–ethyl acetate (95:5, *v*/*v*) mobile phase. Essential oil stock solutions (250 mg/mL) were applied in volumes of 1, 2, and 3 µL, corresponding to 0.25, 0.50, and 0.75 mg per spot. Due to excessive spot broadening observed during preliminary experiments, ajowan and oregano oils were applied in reduced volumes (0.5 and 1 µL; 0.125 and 0.25 mg per spot) in the bioautography assay. Thyme EO was also applied in volumes of 0.5 and 1 µL, corresponding to 0.125 and 0.25 mg per spot. The qualities of the separations were visualised using (**A**) UV illumination (254 nm) and (**B**) vanillin-sulphuric acid reagent-based development. Antibacterially active compounds were visualised using (**C**) bioautography with 3-(4,5-dimethylthiazol-2-yl)-2,5-diphenyltetrazolium bromide (MTT) staining. Inhibition zones were observed for ajowan EO (Rf = 0.56 and Rf = 0.34), oregano EO (Rf = 0.51 and Rf = 0.31), clove EO (Rf = 0.50 and Rf = 0.20), and lime EO (Rf = 0.35 and Rf = 0.22). By contrast, palmarosa EO produced several minor inhibition zones distributed across the chromatogram, with detectable activity at Rf values of 0.65, 0.45, 0.35, and 0.25. Thyme EO also exhibited a prominent inhibition zone at Rf = 0.51, corresponding to thymol, the major constituent of the investigated EO.

The antibacterial activity of the essential oils under investigation was evaluated using thin-layer chromatography coupled with direct bioautography against ESBL-producing *Escherichia coli*. Chromatographic separation made it possible to visualise the individual constituents under UV illumination and after derivatisation with a vanillin-sulfuric acid reagent. The resulting bioautographic plates revealed the antibacterial activity of the separated compounds (Figure 9).

Of the tested EOs tested, ajowan (*Trachyspermum ammi*) and oregano (*Origanum vulgare*) exhibited the most potent antibacterial activity, as demonstrated by the formation of clear inhibition zones on the bioautographic plates. A prominent inhibition zone was observed for ajowan EO at Rf = 0.56, accompanied by a weaker zone at Rf = 0.34. Similarly, oregano EO produced distinct inhibition zones at Rf = 0.51 and Rf = 0.31. Clove EO (*Syzygium aromaticum*) also exhibited antibacterial activities, with inhibition zones detected at Rf = 0.50 and Rf = 0.20. Lime EO (*Citrus aurantifolia*) demonstrated weaker antibacterial activity, with inhibition zones observed at Rf = 0.35 and Rf = 0.22. By contrast, palmarosa EO (*Cymbopogon martinii*) produced several minor inhibition zones distributed across the chromatogram, with detectable activity at Rf values of 0.65, 0.45, 0.35, and 0.25.

Thyme essential oil also exhibited a prominent inhibition zone at Rf = 0.51, corresponding to thymol, the major constituent of the investigated essential oil. Another zone of inhibition is observed at Rf = 0.2.

Overall, bioautographic analysis indicated that antibacterial activity was associated with specific chromatographically separated fractions of the EOs, rather than their entire compositions.

### 2.9. Bioautographic Evaluation of Major Essential Oil Constituents

To identify the compounds responsible for the antibacterial activity of the essential oils, the major constituents were analysed using the same thin-layer chromatography (TLC)–direct bioautography procedure.

**Figure 10:**
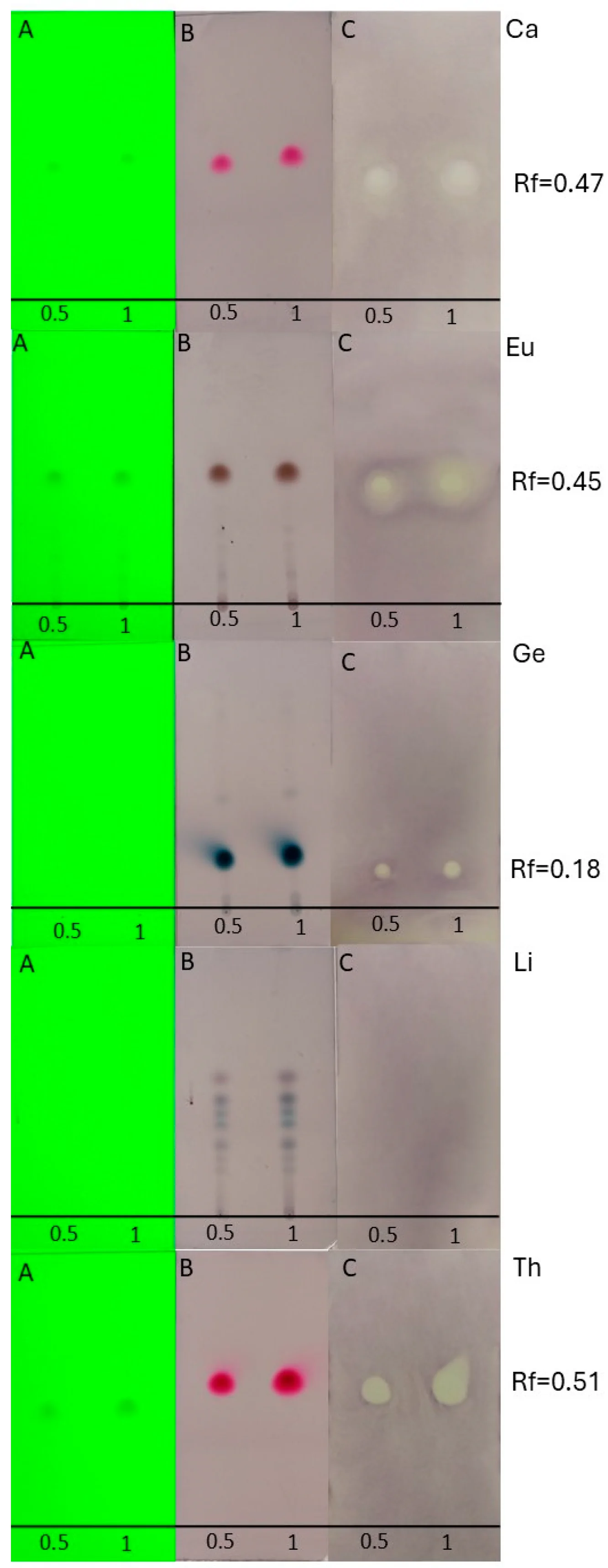
The antibacterial activities of the major compounds of selected EOs were confirmed using (**A**,**B**) thin-layer chromatography and (**C**) direct bioautography against ESBL-producing *Escherichia coli*. The reference compounds, including carvacrol (Ca), eugenol (Eu), geraniol (Ge), limonene (Li), and thymol (Th), were separated on silica gel 60 F254 TLC plates using toluene–ethyl acetate (95:5, *v*/*v*) as the mobile phase. Clear inhibition zones were observed for thymol (Rf = 0.51), eugenol (Rf = 0.45), carvacrol (Rf = 0.47), and geraniol (Rf = 0.18). Limonene showed minimal or no detectable antibacterial activity under the applied conditions.

Clear inhibition zones were observed for thymol (Rf = 0.51) and eugenol (Rf = 0.45), indicating their strong antibacterial activity against the tested ESBL-producing *E. coli* strain. Carvacrol also exhibited antibacterial activity, with inhibition detected at Rf = 0.47. In contrast, geraniol (Rf = 0.18) produced only weak inhibition zones, and limonene showed minimal or no detectable antibacterial activity under the applied conditions (Figure 10).

Comparing the chromatographic and bioautographic profiles of the EOs with those of the reference compounds suggested that the antibacterial activity observed in ajowan and oregano EOs is largely due to the presence of thymol and carvacrol, respectively. Consequently, the antibacterial activity of clove EO corresponds well with the presence of eugenol.

These results demonstrate that the antimicrobial activity of the tested EOs against ESBL-producing *E. coli* is primarily associated with specific phenolic monoterpenes present as major constituents.

## 3. Discussion

ESBL-producing *E. coli* poses a global challenge to human health. Their presence in animal husbandry and slaughterhouses, and their capability to survive on meat surfaces, highlights the need to disinfect surfaces. As well as traditional chemical disinfectants, essential-oil-based products offer new eco-friendly options. The antibacterial efficacy of the nearly 50 EOs from the 60, applied at either 5% or 1% concentrations, has suggested that most of the tested EOs are potent oils for practical applications (Figure 1). Based on the efficacy results, it can be concluded that there were no significant differences in the efficacy of the EOs when applied at different temperatures, including 14 °C, 23 °C, and 37 °C. The balanced antibacterial effect observed for the EOs tested on ESBL-producing *E. coli* at three temperatures indicated that they are suitable candidates throughout the food chain, typically within the range of 4–23 °C. This is an important finding as recent studies have revealed temperature-dependent antibacterial actions of EOs on Salmonella isolates and, even more so, on *Listeria monocytogenes* [24,25], although most studies have only tested the antibacterial efficacies of EOs at 37 °C [26,27,28]. While 4 °C would have been relevant for the tests, none of the ESBL-producing *E. coli* isolates exhibited significant growth at this low temperature, making it difficult to interpret the results reliably [29]. This is why biofilm formation tests were only performed at 14 °C, 23 °C, and 37 °C.

From a practical perspective, the implementation of biofilm inhibition tests is also justified since biofilm formation, as a survival strategy, enhances the spread of bacteria on both biotic and abiotic surfaces [30,31]. Recent studies have reported that biofilm formation can be strongly influenced by the carrier medium [32], which we also confirmed. However, temperature was also revealed to be a crucial factor in this process (Figure 2). Recent studies have demonstrated that 50–60% of the ESBL-producing *E. coli* isolates could be strong biofilm formers, depending on whether the bacteria were isolated from the urinary tract infections, pigs, or other sources [33,34,35]. Other studies characterised ESBL-producing *E. coli* as average biofilm formers [36,37], but it was generally stated that strains belonging to phylogroups A and B2 seemed to have lower ability to form biofilms compared to other phylogroups (B1, D, E, and F) [36,38,39]. Within the scope of this study, we did not investigate the reason for the outstanding biofilm formation capacity of the pig isolate 23R20 (Figure 3), but this characteristic actually served as a good model for further experiments aimed at studying the biofilm inhibitory and degradation potentials of 60 different EOs.

The more or less dense but scattered location of the isolate 23R20 on the stainless steel surface, revealed by scanning electron microscope analysis (Figure 4), did not show any classical biofilm formation but clearly demonstrated that this bacterium isolate was able to adhere widely to the metallic surface commonly found in slaughterhouse environments [40]. Its persistence for extended periods of time on metallic surfaces could serve as a significant source of cross-contamination along the slaughter line [41], as well as healthcare settings [42], given that stainless steel is also a frequently used material in hospitals.

Due to its significance in bacterial survival on solid surfaces, the inhibition and degradation of biofilms is particularly important. The extended biofilm-inhibitory efficacy of clove, oregano, thyme, petitgrain, palmarosa, orange, cinnamon, and ajowan oil at lower temperature (14 °C, Figure 5) strongly suggests their practical relevance as potential disinfecting agents. However, eliminating biofilms poses a greater challenge for EOs, since not all of the aforementioned EOs with strong inhibitory potential were able to fully eliminate mature biofilms (Figure 6). The effect of the carrier medium on the efficacy of EOs is a significant factor that must be considered in future applications. The differences in antibacterial effect in MH-II and BHI could primarily be driven by the medium’s composition, which can support or hinder the effectiveness of an antimicrobial agent. This phenomenon has been described, especially in the case of metal ions such as silver and copper, which exhibit higher activity in minimal media than in complex, nutrient-rich media. This was recently investigated, with significant differences found in the biofilm-degrading efficacy of EOs when applied in tryptic soy broth or an artificial in vitro wound milieu [43]. One possible explanation is that the solubilisation of the EOs depends on the medium. Conversely, the presenting medium strongly influences the membrane fatty acid profile of bacterial membranes, an important factor in EO sensitivity [44,45,46].

The strong, albeit somewhat distinct, antibacterial effects of ajowan, clove, thyme, lime, palmarosa, and oregano prompted us to investigate the differences in their actions. Some characteristic morphological changes in the 23R20 bacterial cells treated in different ways suggested different modes of action for these EOs. Changes in size and morphology, such as elongation or shortening, observed in *Campylobacter jejuni* cells in previous studies suggested that clove and peppermint oils can influence the cellular skeleton of these bacteria [47,48]. The shortened structures observed in cases of clove could suggest a similar mechanism, while the fused, elongated cell structure, resulting from ajowan treatment, which is completely different to the classical *E. coli* coccobacilli shape, suggested the involvement of another background process. These occurrences could be more clearly observed after a longer incubation period, except in the case of lime, where regained cell morphology by the 24th hour suggested a cellular process through which the cell could regain its typical morphology. In this case, the lime oil was present at a subinhibitory concentration, and the 23R20 ESBL-producing *E. coli* was stressed by the lime but eventually overcame the existing stress condition.

The punctured phenotype of the affected individual cells in the case of the oregano and thyme treatments (Figure 6) suggested a more aggressive effect, highlighting a similar mode of action for these EOs. This is the classical membrane-disrupting mode of action of EOs, which was the most pronounced in their case [21]. Since thymol and carvacrol are common components of these EOs, one or both of these compounds may be responsible for these effects [49]. In addition, their precursors, such as *p*-cymene and gamma-terpinene, are also present in larger quantities. However, although both of these compounds have the ability to interact with the cell membrane (especially *p*-cymene), they mostly cause increased permeability rather than destruction, which would have a significant impact on bacterial cell integrity.

The amorphous, ‘smeared’ material visible around the bacterial cells, particularly in the case of ajowan, may be the result of stress-induced EPS overproduction (Figure 7). Ajowan is not a frequently studied EO, but if it upregulates LOS synthesis in *E. coli*, this is somewhat contradictory to previous studies that showed that clove and peppermint upregulate *wlaB*, an LOS biosynthesis gene, in *Campylobacter jejuni* [47,48]. This highlights the differential modes of action of EOs and their different effects on various bacteria, which must also be considered from a practical point of view.

## 4. Materials and Methods

### 4.1. Bacterial Strains, Media

Twenty different ESBL-producing *Escherichia coli* isolates were used in this study. The bacteria were isolated from pig farms and urinary tract infections in Hungary and Germany [50]. Luria–Bertani (LB) agar plates were used for maintenance, and prior to the experiments, single colonies were picked up and inoculated in 5 mL of LB broth. After 14 h of growth at 120 rpm, at 37 °C, optical densities of the bacterium suspensions were synchronised of 0.2 at 630 (approximately 10^8^ CFU/mL). These suspensions were then used for all subsequent experiments, including the drop plate assay, biofilm formation, and inhibition assays. For the biofilm assays, Müller–Hinton II (MH-II; Biolab Co., Budapest, Hungary) and Brain Heart Infusion (BHI; Biolab Co., Hungary) were used.

### 4.2. Essential Oils

Sixty essential oils were used in this study either in concentrated or diluted form, as indicated in the relevant sections. One and five percent emulsions were prepared from the concentrated EOs in 1% Tween 20 solution. Sterility tests were regularly performed on the EO suspensions during the experiments. All EOs were extracted by steam distillation and purchased from the qualified provider, A.G. Industries (Noida, UP, India). A complete list of the used EOs can be found in Supplementary Table S1.

### 4.3. Antibacterial Testing of ESSENTIAL Oils—Drop Plate Method

The antibacterial activity of 60 essential oils was evaluated using the drop plate method as previously described [19,20]. One hundred microliters of the optically synchronised bacterial suspensions were spread evenly onto Luria–Bertani agar plates using a sterile glass spreader to create uniform bacterial lawns. Then, aliquots of 5 µL from 1% and 5% EO solutions were spotted onto the agar surface. The plates were then incubated at 14 °C, 23 °C, or 37 °C. After 24 h, inhibition zones were recorded for plates incubated at 23 °C and 37 °C, while plates kept at 14 °C were evaluated after 72 h [19]. The inhibition zone diameters were measured and expressed in millimetres.

### 4.4. Biofilm-Formation Tests

The biofilm-forming capacities of the 20 ESBL-producing *E. coli* isolates were evaluated at 14 °C, 23 °C, and 37 °C using 96-well polystyrene tissue culture plates (Sarstedt, Nümbrecht, Germany) featuring three surface types with different adhesion properties. Adherent (code: 83.3924), complex adherent (code: 83.3924.300), and suspension cell surfaces (code: 83.3924.500) were used, referred to below as A, CAC, and SC, respectively. In addition, samples from stainless steel mesh (SSM) (dimensions: 0.27 × 0.27 × 0.16; area: 25 mm^2^; Metmark Kft, Szekszárd, Hungary) were also used for the biofilm tests.

The experiments were performed in three different carrier media: LB, MH-II, and BHI. For the tests, overnight bacterial cultures (adjusted to an OD_630_ of 0.2) were diluted 1:100 in LB, MH-II, or BHI media. From each dilution, 200 µL was added to the wells of 96-well plates. Depending on the temperature, the plates were incubated for 18 h at 23 °C and 37 °C, or for 2 days at 14 °C. The experiment was performed with technical triplicates.

After incubation, the non-adherent bacteria were carefully removed, and the wells were rinsed three times with PBS. The adherent (sessile) cells were then fixed for two minutes using 200 µL of a 2% formalin solution (prepared by mixing 28.6 mL of 35% formalin with 471.4 mL of PBS). After disposing of the fixative, the plates were dried at 37 °C for two hours. The plates were stained with a 0.13% crystal violet solution (consisting of 14.29 mL of 35% formalin, 234.41 mL of PBS, 1.302 mL of 96% ethanol, and 0.325 g of crystal violet) for 20 min, followed by three washes of PBS. The bound dye was then solubilized with 200 µL of 1% SDS solution (a 1:1 mixture of 50% ethanol and PBS) for two hours. Absorption was measured at 630 nm using an AMR-100T multimode microplate reader (Allsheng, Hangzhou, China) [51].

To test for biofilm formation on the SSC surface, the steel nets were first disinfected in 96% ethanol for 30 min and then sterilised in an autoclave. The sterilised SSC membranes were placed in the wells of 24-well plates and incubated under the same conditions as those described for the polystyrene plates. After incubation, the nets were washed three times with PBS and fixed in 2% formalin as described above. The biofilms on the SSMs were then analysed by using a scanning electron microscope.

### 4.5. Scanning Electron Microscope (SEM) Analysis

Scanning electron microscopy was used to visualise the formation of biofilm by ESBL-producing *E. coli* strain 23R20 on the surface of stainless steel as well as the effects of ajwain, clove, lime oil (as per BP), palmarosa, oregano, and thyme on the mature biofilm. To achieve this, stainless steel was cut into 1 cm by 1 cm pieces. Each piece was soaked in 95% ethanol for a day and then autoclaved the next day. After sterilisation, each piece was dropped into a separate well of a 24-well polystyrene plate.

The same conditions as described above were used for biofilm formation—more precisely, at three temperatures, 14 °C, 23 °C, and 37 °C, and with three carrier media, LB, MH-II, and BHI. The incubation times also varied: one day at 23 °C and 37 °C, two days at 14 °C, and six weeks at 4 °C. For each test, 990 µL of the chosen culture medium was added to each well, followed by 10 µL of a bacterial suspension set at 0.2 OD_630_ nm.

After incubation, the stainless-steel pieces were placed in the plates and washed three times with 1 mL PBS. They were then fixed with 2.5% glutaraldehyde for two hours. The samples were then dehydrated, using ethanol at 50%, 80%, and 96% for 30 min each.

SEM analysis was also used to visualise the effect of the antibacterial EO treatment on cell morphology. To do this, 1 mL of the cultured strain 23R20 (OD_620_ of 0.2) was centrifuged, after which the sedimented bacterial pellets were resuspended in 100 µL of pure essential oil. The mixture was then incubated for either 1 or 24 h before being fixed with 2.5% glutaraldehyde for 2 hours. After dehydration, the samples were rehydrated with 50%, 80%, and 96% ethanol treatments, each lasting for 10 min. Then, the samples were dried using 50% and 100% hexamethyldisilazane (HMDS) for 30 min on clean microscopic slides. The bacterial cells were then fixed onto aluminium plugs with a thin gold layer using a JEOL JFC-1300 coater (JEOL, Tokyo, Japan). Images were captured on a JEOL JSM-IT500HR SEM (JEOL, Tokyo, Japan) in secondary electron mode at different magnifications, with an acceleration voltage of 5 kV and a probe current of 45 kV.

### 4.6. Testing the Biofilm Inhibitory and Disruptive Effects of EOs

To test the inhibitory efficacy of essential oils on biofilms, the ESBL-producing *E. coli* strain 23R20 was selected, and the experiment was conducted as described in Section 4.4. This exposed the bacteria to the EOs. All experiments were conducted on adhesion cell surfaces (Sarstedt, Nümbrecht, Germany; 83.3924.300) and incubated either at 23 °C or 37 °C for 24 h, or at 14 °C for 48 h. After incubation, viable cell numbers were determined by serial dilution. Biofilm matrix formation on parallel plates was determined using crystal violet staining, as previously described (Section 4.4). Following the standard MIC/MBC protocol guidelines (e.g., CLSI/EUCAST principles), CFU plating was conducted exclusively for wells that showed visible clearing (lack of turbidity/opalization) to assess whether the observed growth inhibition (biofilm inhibition also in this case) was bactericidal or bacteriostatic. Wells that remained visibly turbid/cloudy were categorised as showing active bacterial growth and, per standard protocol, were not subjected to subculturing/CFU determination.

For the mature biofilm degradation cell assay, strain 23R20 was allowed to form biofilms for one day at 23 °C and 37 °C, two days at 14 °C, and six weeks at 4 °C (see Section 4.4). After this time, the growth medium was removed, and the non-adherent cells were washed out with PBS. Next, 200 µL of sterile fresh MH-II/BHI medium containing 0.1% EOs was added to each well. The plates were then incubated for 24 h at various temperatures. After incubation, the matrix was fixed, stained with crystal violet, and measured as previously described. To check for bactericidal effects, samples were taken from the MH-II/BHI wells, after which the number of colony-forming units (CFUs) was determined. The experiment was repeated three times at three different time points.

### 4.7. Thin-Layer Chromatographic Separation of Essential Oils

The antibacterial activity of a selection of essential oils was investigated using thin-layer chromatography combined with direct bioautography (TLC-DB). These oils were *Trachyspermum ammi* (ajowan), *Syzygium aromaticum* (clove), *Citrus aurantifolia* (lime), *Origanum vulgare* (oregano), and *Cymbopogon martinii* (palmarosa).

Chromatographic separation was performed on 5 × 10 cm aluminium-backed silica gel 60 F254 TLC plates (Merck, Darmstadt, Germany). The EOs were dissolved in absolute ethanol to obtain stock solutions with a concentration of 250 mg/mL. Using micropipettes, aliquots of 1, 2, and 3 µL were applied to the plates, corresponding to 0.25, 0.50, and 0.75 mg of EO on the chromatographic layer.

During preliminary experiments, the application of 3 µL resulted in excessive spot broadening and zone overlap ajowan and oregano EOs, hindering the interpretation of the bioautographic inhibition zones. Therefore, the applied volumes for these two oils were reduced to 0.5 and 1 µL (corresponding to 0.125 and 0.25 mg of EO on the plate) in the final bioautography experiments. For chromatographic visualisation (UV detection and vanillin-sulfuric acid derivatisation), however, the original application volumes (1–3 µL) were retained to allow direct comparison with the other EOs investigated.

The chromatograms were developed using a toluene-ethyl acetate solvent system (95:5, *v*/*v*) in a pre-saturated twin-trough chamber (Camag, Muttenz, Switzerland) at room temperature (approximately 22 °C). After development, the plates were dried at 90 °C for 5 min to remove any residual solvent. Chemical visualisation of the separated compounds was performed on replicate plates using an ethanolic vanillin–sulfuric acid reagent under UV light at 254 nm. Plates intended for bioautography were not derivatised to avoid interference with microbial detection. The experiment was repeated three times, performed in three different time points.

### 4.8. Direct Bioautography Assay

The direct bioautography technique was used to evaluate antibacterial activity against an ESBL-producing *Escherichia coli* strain. A bacterial suspension was prepared and adjusted to an optical density of 0.2 at 630 to ensure a standardised inoculum density. The developed TLC plates were then immersed in 100 mL of the bacterial suspension for 30 s to allow for uniform coverage of the chromatographic surface by bacteria. The plates were then incubated at 37 °C for 10 min.

To visualise bacterial metabolism, the plates were immersed in an aqueous solution of 3-(4,5-dimethylthiazol-2-yl)-2,5-diphenyltetrazolium bromide (MTT) (Sigma-Aldrich, Darmstadt, Germany) for around 10 s. After drying, the plates were incubated again at 37 °C to enable metabolically active bacterial cells to reduce MTT. Viable bacterial growth resulted in the formation of purple formazan, whereas colourless inhibition zones indicated antibacterial activity of the chromatographically separated compounds. The inhibition zones became clearly visible after approximately 3 h of incubation. The study was repeated three times, performed at three different time points.

### 4.9. Bioautographic Evaluation of Major Essential Oil Constituents

To identify the compounds responsible for the observed antibacterial activity in the essential oils, the major constituents were evaluated using the same TLC-direct bioautography procedure. Reference standards were dissolved in absolute ethanol to obtain stock solutions at a concentration of 20 mg/mL. Using micropipettes, aliquots of 0.5 and 1 µL were applied to the TLC plates, corresponding to the deposition of 0.01 mg and 0.02 mg of compound, respectively, on the chromatographic layer. The compounds under investigation included limonene, eugenol, geraniol, thymol, and carvacrol (Sigma-Aldrich Ltd., St. Louis, MI, USA). Chromatographic separation was carried out under the same conditions as for the EOs, using silica gel TLC plates and a toluene-ethyl acetate (95:5, *v*/*v*) mobile phase.

Following chromatographic development, the plates underwent the same direct bioautography assay against ESBL-producing *Escherichia coli*. This approach enabled the correlation of antibacterial inhibition zones with the presence of individual constituents in the EOs. The experiment was repeated three times.

### 4.10. Statistical Analysis

To visualise the results, SRplot (https://www.bioinformatics.com.cn/srplot, accessed on 1 May 2026) was used to create the balloon plots, while heatmaps were generated using the website https://www.chiplot.online/circle_heatmap.html. For statistical analyses, the JASP 0.18.3.0 program was used. In the statistical analysis of general biofilm formation, first, the homogeneity of variances using the Levene test was used. Here, the *p*-value (*p* < 0.001) led to the rejection of the null hypothesis. The Shapiro–Wilk p-value was *p* < 0.001 for each strain, indicating that our data do not follow a normal distribution. Therefore, the Kruskal–Wallis test was applied, where a *p*-value of *p* < 0.001 indicates a significant difference.

## 5. Conclusions

Essential oils derived from ajowan, clove, lime, palmarosa, and oregano are not only antibacterial but also can prevent biofilm formation and destroy mature biofilms. Since the efficacy of EOs is greatly affected by the medium, this issue must be given special attention when considering practical applications. The different morphological outcomes following treatment with these six EOs suggest that they have different mechanisms of action. Furthermore, the comparison of the antibacterial efficacies and mode of actions of different compounds with antibacterial activities could be exploited in the development of future therapeutic agents or identify more precise therapeutic targets in bacteria.

## Figures and Tables

**Table 1:** Stepanovic’s biofilm formation groups. Isolate 23R20 demonstrated a highly stable biofilm-forming phenotype across the tested environmental conditions. It produced a strong biofilm in 26 of the 27 environmental combinations and a moderate biofilm in only one combination, with no weak or non-biofilm-forming phenotype observed. This high consistency indicates that the strong biofilm-forming capacity of 23R20 was largely maintained across the tested temperatures, growth media, and surfaces. Overall, 23R20 showed the most consistent strong biofilm-forming phenotype among the tested isolates and was classified as a stable biofilm former (NNK1).

Strains	Strong	Middle	Weak	Without Biofilm	All Combination
23R20	26	1	0	0	27
NNK1	8	7	12	0	27
1/13x	4	4	18	1	27
szp2	4	3	11	9	27
4011	4	2	12	9	27
4187	3	4	17	3	27
4159	2	10	7	8	27
NNK2	2	5	10	10	27
23B6X	2	0	12	13	27
TT70035	1	3	11	12	27
SM09658	1	2	9	15	27
JM143x1	1	1	5	20	27
SR00754	0	4	9	14	27
JM233	0	2	12	13	27
szp3	0	2	10	15	27
szp1	0	1	12	14	27
23B43X	0	0	20	7	27
SR01077	0	0	15	12	27
4/40	0	0	8	19	27
LBR49/3	0	0	6	21	27

## Data Availability

All data are available in the manuscript.

## Supplementary Materials

The following supporting information can be downloaded at: https://www.mdpi.com/article/10.3390/antibiotics15101022/s1, Table S1: List of applied essential oils.
